# A treatment strategy with nifedipine versus labetalol for women with pregnancy hypertension: study protocol for a randomised controlled trial (Giant PANDA)

**DOI:** 10.1186/s13063-023-07582-9

**Published:** 2023-09-12

**Authors:** Danielle Ashworth, Cheryl Battersby, Debra Bick, Marcus Green, Pollyanna Hardy, Lisa Leighton, Laura A. Magee, Alisha Maher, Richard J. McManus, Catherine Moakes, R. Katie Morris, Catherine Nelson-Piercy, Jenie Sparkes, Oliver Rivero-Arias, Andrew Webb, Hannah Wilson, Jenny Myers, Lucy C. Chappell

**Affiliations:** 1 Department of Women and Children’s Health, School of Life Course Sciences, King’s College London, London, UK; 2 Neonatal Medicine, School of Public Health, Faculty of Medicine, Imperial College London, London, UK; 3 Warwick Clinical Trials Unit, Warwick Medical School, University of Warwick, Coventry, UK; 4 Action On Pre-Eclampsia, Evesham, UK; 5 National Perinatal Epidemiology Unit Clinical Trials Unit, Nuffield Department of Population Health, University of Oxford, Oxford, UK; 6 Birmingham Clinical Trials Unit, Institute of Applied Health Research, University of Birmingham, Birmingham, UK; 7 Institute of Women and Children’s Health, King’s College London, London, UK; 8 Nuffield Department of Primary Care Health Sciences, University of Oxford, Oxford, UK; 9 Obstetric Medicine, Guy’s and St Thomas’ Hospitals NHS Trust, London, UK; 10 Clinical Pharmacology, School of Cardiovascular and Metabolic Medicine & Sciences, King’s College London British Heart Foundation Centre of Research Excellence, London, UK; 11 Faculty of Biology, Medicine and Health, The University of Manchester, Manchester, UK

**Keywords:** Hypertension, Pregnancy, Randomised controlled trial, Antihypertensives, Blood pressure

## Abstract

**Background:**

Approximately one in ten women have high blood pressure during pregnancy. Hypertension is associated with adverse maternal and perinatal outcomes, and as treatment improves maternal outcomes, antihypertensive treatment is recommended. Previous trials have been unable to provide a definitive answer on which antihypertensive treatment is associated with optimal maternal and neonatal outcomes and the need for robust evidence evaluating maternal and infant benefits and risks remains an important, unanswered question for research and clinical communities.

**Methods:**

The Giant PANDA study is a pragmatic, open-label, multicentre, randomised controlled trial of a treatment initiation strategy with nifedipine (calcium channel blocker), versus labetalol (mixed alpha/beta blocker) in 2300 women with pregnancy hypertension. The primary objective is to evaluate if treatment with nifedipine compared to labetalol in women with pregnancy hypertension reduces severe maternal hypertension without increasing fetal or neonatal death or neonatal unit admission. Subgroup analyses will be undertaken by hypertension type (chronic, gestational, pre-eclampsia), diabetes (yes, no), singleton (yes, no), self-reported ethnicity (Black, all other), and gestational age at randomisation categories (11 + 0 to 19 + 6, 20 + 0 to 27 + 6, 28 + 0 to 34 + 6 weeks). A cost-effectiveness analysis using an NHS perspective will be undertaken using a cost-consequence analysis up to postnatal hospital discharge and an extrapolation exercise with a lifetime horizon conditional on the results of the cost-consequence analysis.

**Discussion:**

This trial aims to address the uncertainty of which antihypertensive treatment is associated with optimal maternal and neonatal outcomes. The trial results are intended to provide definitive evidence to inform guidelines and linked, shared decision-making tools, thus influencing clinical practice.

**Trial registration:**

EudraCT number: 2020–003410-12, ISRCTN: 12,792,616 registered on 18 November 2020.

**Supplementary Information:**

The online version contains supplementary material available at 10.1186/s13063-023-07582-9.

## Administrative information

Note: the numbers in curly brackets in this protocol refer to SPIRIT checklist item numbers. The order of the items has been modified to group similar items (see http://www.equator-network.org/reporting-guidelines/spirit-2013-statement-defining-standard-protocol-items-for-clinical-trials/).

**Table:** 

Title {1}	A treatment strategy with nifedipine versus labetalol for women with pregnancy hypertension: study protocol for a randomised controlled trial (Giant PANDA)
Trial registration {2a and 2b}.	EudraCT number: 2020–003410-12 ISRCTN: 12,792,616
Protocol version {3}	Version 1.1, 11/01/2021
Funding	National Institute for Health and Care Research Health Technology Assessment programme
Author details {5a}	Danielle Ashworth^1^ Cheryl Battersby^2^ Debra Bick^3^ Marcus Green^4^ Pollyanna Hardy^5^ Lisa Leighton^6^ Laura A Magee^7^ Alisha Maher^6^ Richard J McManus^8^ Catherine Moakes^6^ R. Katie Morris^6^ Catherine Nelson-Piercy^9^ Jenie Sparkes^1^ Oliver Rivero-Arias^5^ Andrew Webb^10^ Hannah Wilson^1^ Jenny Myers^11^ Lucy C Chappell^1^ 1- Department of Women and Children’s Health, School of Life Course Sciences, King’s College London 2- Neonatal Medicine, School of Public Health, Faculty of Medicine, Imperial College London 3- Warwick Clinical Trials Unit, Warwick Medical School, University of Warwick 4- Action on Pre-eclampsia 5- National Perinatal Epidemiology Unit Clinical Trials Unit, Nuffield Department of Population Health, University of Oxford 6- Birmingham Clinical Trials Unit, Institute of Applied Health Research, University of Birmingham 7- Institute of Women and Children’s Health, King’s College London, UK 8- Nuffield Department of Primary Care Health Sciences, University of Oxford 9- Obstetric Medicine, Guy’s and St Thomas’ Hospitals NHS Trust 10- Clinical Pharmacology, School of Cardiovascular and Metabolic Medicine & Sciences, Kings College London British Heart Foundation Centre of Research Excellence 11- Faculty of Biology, Medicine and Health, The University of Manchester
Name and contact information for the trial sponsor {5b}	University of Birmingham, researchgovernance@contacts.bham.ac.uk
Role of sponsor {5c}	The study funder and sponsor have had no input into the study design, report writing or the decision to submit for publication (beyond usual governance activities).

## Introduction

### Background and rationale {6a}

Approximately 1 in 10 women have hypertension or high blood pressure in pregnancy. This includes chronic (pre-existing, typically essential) and gestational (new from 20 weeks’ gestation) hypertension and pre-eclampsia (hypertension with additional features of maternal multi-organ or uteroplacental involvement). Hypertensive disorders of pregnancy, in particular pre-eclampsia, are associated with substantial maternal and perinatal morbidity [1–3] and mortality [4].

Treatment with antihypertensives is typically offered to pregnant women to lower blood pressure and prevent progression to severe hypertension, known to be associated with adverse maternal and perinatal outcomes.

The 2018 Cochrane systematic review of antihypertensive trials in pregnancy included data from 58 trials and 5909 women. It concluded that treatment benefit is such that no further research is needed on treatment versus no treatment, noting that the use of any antihypertensive drug (versus placebo or no antihypertensive drug) halves the risk of developing severe hypertension (20 trials, 2558 women; risk ratio (RR) 0.49; 95% confidence interval (CI) 0.40–0.60) [5]. A trial published since this systematic review demonstrated a significant reduction in pre-eclampsia (RR, 0.79; 95% CI, 0.69 to 0.89) and preterm birth (RR, 0.87; 95% CI, 0.77 to 0.99) in women actively treated for mild chronic hypertension compared to the control group [6]. It further concluded that future research should be high-quality, large-sized, randomised controlled trials focused on the head-to-head assessment of antihypertensive drugs. Only two trials [7, 8], with a total of 354 women, compared the top two antihypertensive treatments recommended across international guidelines, namely labetalol (a mixed alpha/beta-adrenoceptor blocker) and nifedipine (calcium channel blocker) [5, 7, 8]. A network meta-analysis, published since the commencement of recruitment to this trial, confirmed the reduction in severe hypertension with both agents versus no therapy and recommended that further information from a variety of sources should be obtained [9]. A feasibility study (not powered for clinical outcomes) found a 7.4 mmHg (− 0.4 to − 14.4) reduction in central aortic pressure with nifedipine (vs. labetalol), but definitive evidence for superiority of one agent over another remains to be established [7, 8].

First-line therapy across international guidelines is typically labetalol or nifedipine [10], with no advice on tailoring of drug therapy for ethnicity, as recommended out of pregnancy [11]. The UK National Institute for Health and Care Excellence (NICE) pregnancy Hypertension guideline advises labetalol as first-line treatment based on its license for use in pregnancy, with a research recommendation to evaluate the effectiveness and safety of antihypertensives (in head-to-head trials) in improving maternal and perinatal outcomes [12].

The Giant Pregnancy ANtihypertensive Drugs: which Agent is best? (PANDA) https://fundingawards.nihr.ac.uk/award/NIHR128721 study aims to compare a treatment strategy of nifedipine versus labetalol in women with pregnancy hypertension. It addresses a research recommendation first published by NICE in 2010 and reiterated in the 2019 update [12]. Our study is also informed by the findings of a four-centre feasibility study (ISRCTN40973936) and developed by a multidisciplinary team with outcomes chosen by patient and public involvement. This study is funded by the National Institute for Health and Care Research Health Technology Assessment programme and has been approved by the Health Research Authority and Medicines and Healthcare products Regulatory Agency, together with NHS Trust Research and Development Offices for each site.

## Objectives {7}

The objective of this study is to address the research question: “In women with pregnancy hypertension (Population), what is the effect of treatment with nifedipine (Intervention) versus labetalol (Comparator) on severe maternal hypertension (Outcome) and a composite of fetal or neonatal death, or neonatal unit admissions (Outcome)?”.

### Objectives

The primary objective is to evaluate if treatment with nifedipine (calcium channel blocker), compared to labetalol (mixed alpha/beta blocker) in women with pregnancy hypertension, reduces severe maternal hypertension without increasing fetal or neonatal death or neonatal unit admission.

The secondary objectives are as follows:

- To investigate the effect of treatment with nifedipine versus labetalol on other maternal and fetal/neonatal outcomes including patient-reported outcome measures.
- To evaluate the cost-effectiveness of nifedipine versus labetalol as antihypertensive drugs from an NHS perspective.

## Trial design {8}

The Giant PANDA study is a pragmatic, parallel-group, open-label, multicentre, two-arm randomised controlled trial of treatment with nifedipine versus labetalol by random allocation (1:1) in women with pregnancy hypertension, with two co-primary outcomes: a maternal outcome assessing superiority and a fetal/neonatal outcome assessing non-inferiority. Women who decline randomisation or are unable to be randomised (meeting any of the exclusion criteria detailed below) are offered participation in an observational study, involving data collection only.

The study is anticipated to take around 48 months to complete. This timeline accounts for study set-up, internal pilot, main trial recruitment, follow-up for all women and babies, data analysis, and write-up. An embedded internal pilot is expected to be run in approximately 17 sites over 10 months to assess recruitment and retention rates, acceptability, and implementation. Provided the pre-specified progression criteria are satisfactorily met, recruitment to the main trial would proceed with no break (or interim data analysis) and data from the pilot and main trial analysed together.

## Methods: participants, interventions, and outcomes

### Study setting {9}

The study is being conducted in around 50 consultant-led maternity units (sites) across the UK with a focus on gaining a geographical breadth of sites (across clinical research networks) and a variety of site sizes and types including academic teaching hospitals and hospitals serving peripheral geographic locations. Study site selection is focussing on ensuring that the research follows patient need, particularly with regard to the ethnic diversity of eligible participants. A full list of study sites can be found on the Giant PANDA website https://www.birmingham.ac.uk/research/bctu/trials/womens/giant-panda/index.aspx.

### Eligibility criteria {10}

#### Inclusion criteria

Women who meet all the following criteria are eligible for enrolment in the randomised trial:

- Pregnancy of between 11 + 0 and 34 + 6 weeks’ gestation inclusive
- Diagnosis of pregnancy hypertension (chronic/gestational hypertension or pre-eclampsia) as defined by the NICE 2019 criteria as systolic blood pressure ≥ 140 and/or diastolic blood pressure ≥ 90 mmHg [12]
- Clinician’s decision to initiate or continue the use of antihypertensive drugs
- Aged 18 years or over
- Able to give informed consent

#### Exclusion criteria

Women will be excluded from participation in the randomised trial if they meet either of the following criteria:

- Contraindication to either labetalol or nifedipine
- Already taking both labetalol and nifedipine and not able to be randomised to a single drug

Women who meet either of the exclusion criteria under, are under 11 weeks’ gestation, or decline randomisation will be offered participation in an observational study, involving data collection only.

### Who will take informed consent? {26a}

Women who meet the inclusion criteria are identified and approached by members of the direct clinical care team or the local maternity research team (who are integrated within the direct clinical care team) through referral letters and/or at antenatal clinics (including at antenatal booking, Early Pregnancy Assessment Unit attendances and Maternity Assessment Unit attendances). Potential participants are provided with information on the study, both verbally from a member of the research team and written via the participant information leaflet. They are given the opportunity to ask any questions they may have and then provided with appropriate time to decide whether they wish to participate. Provided the woman is happy to participate, an appropriately trained healthcare professional (who is Good Clinical Practice (GCP) trained and designated to take consent by the site principal investigator) obtains informed consent before performing any study-related procedures. For trial participants, this follows confirmation of eligibility by a medically qualified individual. Study participation and information on the prescription of the study antihypertensive drug is documented in the handheld paper or electronic maternity record as in usual clinical care, as the standard way of communicating with general practitioners and other relevant healthcare professionals.

### Additional consent provisions for collection and use of participant data and biological specimens {26b}

The consent form includes a statement explaining that direct access to maternal and infant medical records is required for participation. Consent for electronic data linkage between routinely collated electronic data records (for the woman and the baby) is also requested to ascertain future outcomes without participant recall, with women free to decline without any impact on their study participation.

Consent for biological specimens is not applicable as no samples are being collected.

## Interventions

### Explanation for the choice of comparators {6b}

The comparator is oral labetalol (no brand specified) which is currently recommended as the first-choice treatment for pregnancy hypertension by NICE guidance in England.

### Intervention description {11a}

The intervention is nifedipine modified-release preparations (no brand specified), which is listed as an acceptable alternative in NICE guidance.

The starting dose for both the intervention and comparator is left to the discretion of the responsible healthcare professional. Sites are asked to follow standard NICE care pathways, for the management of pregnancy hypertension [12]. Apart from the trial treatment allocated at randomisation, all other aspects of clinical management are entirely at the discretion of the local healthcare team. This includes any up- or down-titration, switching, or adding to the randomised antihypertensive drug ensuring that women are effectively and safely treated. All aspects of both interventions are open label.

### Criteria for discontinuing or modifying allocated interventions {11b}

Discontinuation of allocated treatment (stopping or switching antihypertensive treatment) is a common part of usual clinical care during pregnancy (either at the request of the woman, or the clinician), and a woman can continue in the study, for collection of perinatal and maternal outcome data, after discontinuation of treatment. Women are able to withdraw consent for further contact at any time without giving a reason and with no effect on their (or their baby’s) ongoing care. Women continue to receive usual clinical care if they withdraw from the study.

### Strategies to improve adherence to interventions {11c}

As a pragmatic trial, standard clinical advice on adherence to antihypertensives is provided by the prescribing healthcare professional with no additional specific trial advice given. Information on women’s adherence to the antihypertensive medication is captured via participant-completed online surveys at 2 weeks after consent and monthly thereafter.

### Relevant concomitant care permitted or prohibited during the trial {11d}

The Giant PANDA study is embedded in usual clinical care and thus woman can receive concomitant care as clinically required and participate in any other observational study. Co-enrolment in other trials is considered, discussed, and agreed upon by the trial management group.

### Provisions for post-trial care {30}

As antihypertensive treatment is commonly switched immediately after birth, outcomes are collected up to primary hospital discharge or 28 days post-birth, whichever occurs soonest. The decision to continue or switch antihypertensive drugs after birth sits entirely with the clinicians within the woman’s usual care team.

### Outcomes {12}

Outcomes are recorded on the web-based trial-specific database through a review of case notes by trained researchers. Outcomes are collected from randomisation (or consent for women in the observational study only) up to primary hospital discharge for each woman or baby post-birth or 28 days post-birth, whichever occurs soonest.

### Co-primary outcomes

#### Primary maternal outcome

The primary maternal outcome is severe hypertension (proportion of days with healthcare professional measured systolic blood pressure reading ≥ 160 mmHg, out of the total number of days with healthcare professional measured blood pressure readings between randomisation and birth).

#### Primary fetal/neonatal outcome

The primary fetal/neonatal outcome is composite outcome of fetal loss before birth or known neonatal death or neonatal unit admission involving separation of the baby from the mother. This outcome includes all babies born to a randomised mother, the denominator being the number of fetuses/infants.

### Secondary outcomes

#### Secondary maternal outcomes

Outcomes indicated by an asterisk (*) will be presented with a treatment effect and confidence intervals (CI). All other outcomes will be presented with summary statistics only.

Up to birth:

- Mean antenatal systolic blood pressure (using the highest systolic blood pressure per day as collected for the primary outcome)*
- Severe maternal hypertension* (defined as any episode of severe maternal hypertension (systolic blood pressure ≥ 160 mmHg between randomisation and birth))
- Mean antenatal diastolic blood pressure (using the highest diastolic blood pressure per day)
- Proportion of days with an antenatal systolic hypertension blood pressure reading ≥ 140 mmHg
- Proportion of days with an antenatal diastolic hypertension blood pressure reading ≥ 90 mmHg
- New diagnosis of pre-eclampsia*
- Diagnosis of eclampsia
- Diagnosis of haemolysis, elevated liver enzymes, low platelet syndrome
- Placental abruption
- Severe maternal morbidity (fullPIERS consensus definition [13])*
- Components of severe maternal morbidity
- Maternal death
- Maternal stroke
- Prescription of additional antihypertensive drug(s)
- Prescription of alternative antihypertensive drug(s)
- Persistence with allocated antihypertensive (time from randomisation to the first discontinuation)
- Discontinued allocated antihypertensive drug*
- Undesirable effects of allocated (and other) antihypertensive drug(s) (number of women* and number of undesirable effects)
- Total number of antenatal hospital inpatient days

Medication-related self-reported outcomes (measured at 2 weeks post-randomisation, if before birth) using validated tools:

- Treatment satisfaction with the allocated antihypertensive drug.
- Beliefs about allocated antihypertensive drug.
- Adherence to allocated (and other) antihypertensive drug(s).

At delivery/birth:

- Indicated delivery (induction of labour or prelabour rupture of membranes with stimulation of labour or pre-labour caesarean section)*
  - ◦ Indication for the onset of birth
- Mode of onset of birth (spontaneous, induction of labour, prelabour rupture of membranes with stimulation of labour, pre-labour caesarean section).

Between birth and primary hospital discharge or 28 days post-birth, whichever occurs sooner:

- New episodes of severe maternal morbidity (fullPIERS consensus definition [13])).
  - ◦ Components of severe maternal morbidity
- Maternal death.

#### Secondary fetal and neonatal outcomes

Between birth and primary hospital discharge or 28 days post-birth, whichever occurs sooner, unless otherwise specified, using the denominator of all fetuses/ infants:

- Fetal loss before 24 + 0 weeks’ gestation.
- Fetal loss ≥ 24 + 0 weeks’ gestation (stillbirth).
- Known early neonatal death (up to 7 days from birth).
- Known late neonatal death (between 7 and up to 28 days from birth).
- Neonatal unit admission (separation of baby from mother)*
  - ◦ Principal recorded indication for neonatal unit admission
  - ◦ Length of stay in the neonatal unit (and level of care)
- Major congenital abnormality as defined by EUROCAT*
- Mode of birth (spontaneous vaginal,* assisted vaginal, caesarean section).
  - ◦ Indication for the mode of birth
- Gestational age at birth*
- Preterm birth (< 37 completed weeks’ gestation).
- Preterm birth (< 32 completed weeks’ gestation).
- Birthweight.
- Birthweight centile*
- Birthweight small for gestational age (< 10th centile for gestational age).
- Umbilical arterial pH < 7 at birth.
- Apgar score at 5 min after delivery.
- Need for additional resuscitation at birth: intubation in the delivery room, resuscitation drugs, or chest compressions.
- Need for respiratory support.
  - ◦ Type of respiratory support needed
- Need for treatment for neonatal hypoglycaemia (in those having blood glucose monitoring) [14]*
  - ◦ Type of treatment for hypoglycaemia
- Lowest blood glucose measurement within the first 48 h after birth.
- Intracranial haemorrhage.
- Neonatal seizures.
- Necrotising enterocolitis.

#### Process outcomes

- Number of babies in whom blood glucose monitoring was indicated at birth.
- Indication for blood glucose monitoring.
- Blood glucose test performed.
- Acceptability of digital data capture method: proportion of completed responses over expected total number (adjusted for the time between enrolment and delivery).

#### Adverse events

- Adverse event recorded (number of women and number of adverse events)
- Adverse event recorded (number of fetuses/neonates and number of adverse events)

#### Health economic maternal outcomes

Up to birth:

- Health-related quality of life (EQ-5D-5L).
- Number of outpatient contacts.
- Hospital inpatient length of stay by the level of care (intensive care unit, high dependency unit, or ward).

Between birth and primary hospital discharge or 28 days post-birth, whichever occurs sooner:

- Hospital inpatient length of stay by the level of care (intensive care unit, high dependency unit, or ward).

#### Health economic neonatal outcomes

Between birth and primary hospital discharge or 28 days post-birth, whichever occurs sooner:

- Hospital inpatient days by the level of care (neonatal intensive care unit, high dependency unit, special care baby unit, and postnatal ward).

### Participant timeline {13}

**Fig. 1:**
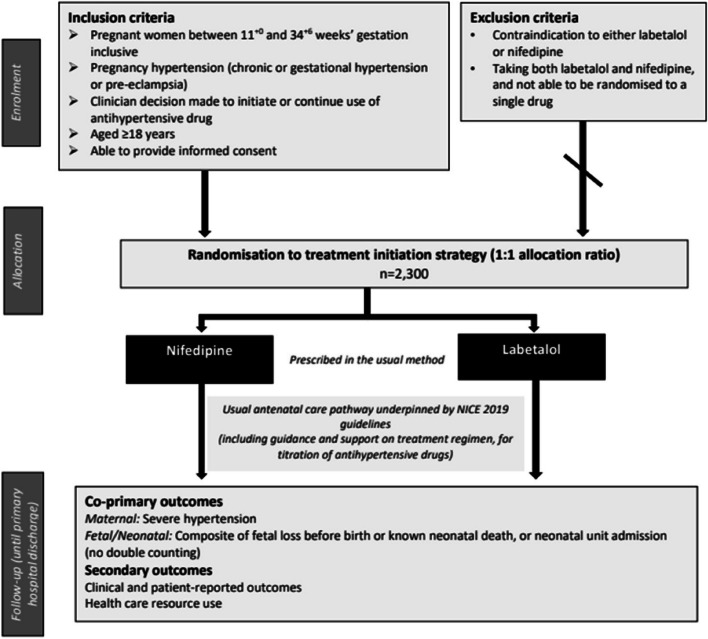
Trial flow diagram

The participant timeline is presented in Fig. 1 and Table 1. As a pragmatic trial, no trial-specific visits are required.

**Table 1:** Schedule of participant enrolment, allocation, and assessment in the trial

	**Enrolment**	**Allocation (trial participants only)**	**Antenatal period**	**At hospital discharge**
Eligibility screen	x			
Informed e-consent	x			
Baseline EQ-5D	x			
Randomisation and prescription of antihypertensive drug (trial participants only)		X		
Two weeks post enrolment contact *(participant completed)*			x	
Six weeks post enrolment contact (four weekly thereafter) *(participant completed)*			x	
Safety reporting (as needed)			x	x
Case note review (safety and outcome data collection)	x (demographic and clinical baseline data)		x	x

### Sample size {14}

#### Non-inferiority hypothesis for the fetal/neonatal co-primary outcome

The sample size calculation was driven by the fetal/neonatal co-primary outcome. Using data from the Control of Hypertension In Pregnancy Study (CHIPS) trial and the PANDA feasibility study [8, 15], the control group event rate for fetal or neonatal death or neonatal unit admission was 25%. A sample size of 2190 babies would have 90% power to detect a non-inferiority margin of 6%, with a 2.5% one-sided significance level. Assuming approximately 1.5% of pregnancies are multi-fetal (Office for National Statistics), a sample size of 2190 women would provide a conservative estimate for the number of babies required to address the hypothesis for the fetal/neonatal co-primary outcome. Due to the anticipated small proportion of multi-fetal pregnancies, this sample size would also allow for the dependence between outcomes for infants from the same pregnancy [16].

#### Superiority hypothesis for the co-primary maternal outcome

For the maternal co-primary outcome, using the PANDA feasibility study data (Webster, Myers et al. 2017), a sample size of 2190 would enable us to detect a 2.3% superiority difference between the mean proportions, equivalent to an effect size of 0.14 of a standard deviation, based on a two-sample *t*-test (5% two-sided alpha, 90% power).

Allowing for up to 5% loss to follow-up, a total sample size of approximately 2300 women, 1150 women per group, will be recruited.

### Recruitment {15}

Women are identified in the antenatal care setting of participating sites, confirmed as eligible to participate (by a medically qualified individual) and provided with information on the study and given appropriate time to decide to participate. An appropriately trained healthcare professional on the delegation log obtains informed electronic consent for participation.

## Assignment of interventions: allocation

### Sequence generation {16a}

Randomisation is managed by a secure online randomisation system, at the Birmingham Clinical Trials Unit. Women are randomised at a 1:1 ratio to either nifedipine or labetalol. A minimisation algorithm is used to ensure balance in the treatment allocation with respect to the recruiting maternity unit, hypertension type (chronic, gestational, pre-eclampsia), diabetic status (yes/no), singleton pregnancy (yes/no), self-reported ethnicity (black, all other), and gestational age at randomisation (11 + 0 to 19 + 6, 20 + 0 to 27 + 6, 28 + 0 to 34 + 6 weeks’). A ‘random element’ is included in the minimisation algorithm, so that each woman has a probability of being randomised to the opposite treatment that they would have otherwise received.

### Concealment mechanism {16b}

As randomisation is performed by a central secure online randomisation system, the allocation sequence is concealed.

### Implementation {16c}

Women are enrolled by an appropriately trained healthcare professional and assigned to either labetalol or nifedipine by the online randomisation system, REDCap. Details of the minimisation algorithm to be used are detailed in the ‘Sequence generation {16a}’ section.

## Assignment of interventions: blinding

### Who will be blinded {17a}

To ensure women are effectively and safely treated, this is an open-label trial with all trial participants, care providers, and outcome assessors unmasked to allocation and therefore aware of allocation to either nifedipine (intervention) or labetalol (comparator). Data analysts are masked to allocation unless required by the data monitoring committee (DMC) for analysis and/or data cleaning.

### Procedure for unblinding if needed {17b}

Unblinding is not needed due to the open-label study design.

## Data collection and management

### Plans for assessment and collection of outcomes {18a}

Trial outcomes are presented in the ‘Outcomes {12}’ section. Before commencing recruitment, each site receives training on the study design, protocol procedures (within the Site Initiation Visit) and data collection procedures (REDCap database training). All members of the site research team are expected to be appropriately trained and work in accordance with the GCP guideline and are required to provide proof of their GCP training and relevant experience. Outcome data are captured from standard maternity notes, by case note review, where clinical data are routinely collected in maternity care during the antenatal period, at birth and up to hospital discharge. Outcomes are captured for each woman or baby up to primary hospital discharge post-birth or at 28 days post-birth, whichever occurs sooner. Data monitoring procedures are covered in the ‘Frequency and plans for auditing trial conduct’ {23} section.

Table 1 described the participant timeline through the study. The participant-reported outcomes are captured within online questionnaires sent to women. The 2-week post consent questionnaire captures the antihypertensive medication prescribed, medication side effects, and four validated questionnaires to capture the health-related quality of life (EuroQol-5 Dimension, EQ-5D-5L [17]), treatment satisfaction (Treatment Satisfaction Questionnaire for Medication, TSQM Version II [18]), cognitive representations of medication (Beliefs about Medicines Questionnaire, BMQ-Specific [19]), and medication adherence (Medication Adherence Report Scale, MARS-5 [20]). At subsequent contact points (6 weeks post consent and four weekly thereafter), antihypertensive medication prescribed and health-related quality of life (EuroQol-5 Dimension, EQ-5D-5L) are captured.

### Plans to promote participant retention and complete follow-up {18b}

Participants are automatically sent a text message inviting them to complete online questionnaires, with an option for telephone completion, at the relevant contact points (Table 1). The site research team monitors the completeness of the 2-week contact questionnaire and contacts participants if questionnaires are missing or incomplete. Women are encouraged to return data even if they stop their allocated treatment.

### Data management {19}

Trial data are collected and stored according to GCP guidelines [21]. Processes to facilitate the accuracy of the trial data are detailed in the data management plan. Coding and validation are agreed upon between the trial manager, statistician, and programmer, and the study database is signed off once the implementation of these has been assured*.*

### Confidentiality {27}

Personal information recorded is regarded as strictly confidential and handled and stored following the Data Protection Act 2018 (and subsequent amendments). Women are always identified using their unique study number. No information by which a woman may be identified is disclosed to any third party other than those directly involved in the treatment of the woman and organisations for which the woman has given explicit consent for data transfer.

### Plans for collection, laboratory evaluation, and storage of biological specimens for genetic or molecular analysis in this trial/future use {33}

No biological samples are being collected.

## Statistical methods

### Statistical methods for primary and secondary outcomes {20a}

A separate statistical analysis plan (see Supplementary material) for the quantitative analysis of the Giant PANDA study will provide a detailed description of the planned statistical analyses. A brief outline is given below. To maintain the rigour of randomisation, the primary analyses will be based on the intention to treat principle for both the maternal co-primary outcome (on a superiority hypothesis) and the fetal/neonatal co-primary outcome (on a non-inferiority hypothesis) and all secondary and safety outcomes. The final analysis will include data items up to and including the primary hospital discharge or 28 days post-birth (whichever occurs sooner) assessment and no further.

All estimates of differences between groups will be presented with two-sided 95% CIs adjusting for the minimisation variables. For fetal/neonatal outcomes, correlation between multiples will be further accounted for in the model. No adjustment for multiple comparisons will be made.

#### Co-primary outcomes

The maternal co-primary outcome (defined in the ‘Outcomes{12}’ section) will be summarised by treatment group using means and standard deviations. A fractional regression model will be used to generate adjusted mean differences (and 95% CIs). The *p*-value relating to the treatment group parameter as generated by the model will be presented.

The fetal/neonatal co-primary outcome (defined in the ‘Outcomes {12}’ section) will be summarised by treatment group using frequencies and percentages. A log-binomial model will be used to generate risk ratios (and 95% CIs). Adjusted risk differences will also be presented (and 95% CIs). A *p*-value will not be presented for this co-primary outcome, as non-inferiority will be assessed based on the upper limit of the 95% CI.

#### Secondary outcomes

The outcomes indicated with an asterisk in the ‘Outcomes {12}’ section will be analysed as described below. Binary secondary outcomes will be analysed as per the fetal/neonatal co-primary outcome. Continuous outcomes which are deemed to be normally distributed will be summarised using means and standard deviations and a linear model will be fitted to generate adjusted mean differences (and 95% CIs). Continuous outcomes which are not deemed to be normally distributed will be summarised using medians and interquartile ranges and unadjusted differences in medians will be produced with 95% CIs. Undesirable effects of allocated antihypertensive drug(s) will be summarised using frequencies and percentages only, unless they occur with a frequency of > 5% in at least one of the treatment groups, whereby they will then be formally analysed as per the binary secondary outcomes described above.

All other secondary outcomes (not indicated with an asterisk in the ‘Outcomes {12}’ section) will be summarised using descriptive statistics only. A separate health economics analysis plan will be produced and will provide a comprehensive description of the planned health economic analyses.

### Interim analyses {21b}

Interim analyses of safety and efficacy outcomes for presentation to the independent DMC will take place during the study. This is likely to include the analysis of the primary and major secondary outcomes and a full assessment of safety (serious adverse events) at least annually. Criteria for stopping or modifying the study based on this information will be ratified by the DMC and documented in the DMC Charter.

### Methods for additional analyses (e.g. subgroup analyses) {20b}

Subgroup analyses will be undertaken on variables used in the minimisation algorithm (detailed in the ‘Sequence generation {16a}’ section) with the exception of the maternity unit and will be limited to the co-primary outcomes only. Tests for statistical heterogeneity (for example by including the treatment group by subgroup interaction parameter in the regression model) will be presented alongside the effect estimate and 95% CI within each subgroup. The results of subgroup analyses will be treated with caution and will be used for hypothesis generation only.

### Methods in analysis to handle protocol non-adherence and any statistical methods to handle missing data {20c}

Every attempt will be made to collect full follow-up data on all participating women and their babies; it is thus anticipated that missing data will be minimal. Women or babies with missing co-primary outcome data will not be included in the primary analysis in the first instance. This presents a risk of bias, and sensitivity analyses will be undertaken to assess the possible impact of this risk.

Since the intention to treat analysis could provide results which are biased towards non-inferiority, for the co-primary outcomes only, sensitivity analyses based on the per-protocol and on-treatment populations will also be performed.

To explore the influence, if any, of blood pressure measurement setting on the maternal co-primary outcomes, an additional sensitivity analysis will include an analysis of all blood pressure readings (in clinic and self-measured, reported in a telephone consultation). A further analysis which only includes women who self-monitor their blood pressure will also be considered.

In addition, since there is a risk of measurement bias for the secondary outcome assessing neonatal hypoglycaemia, we will perform a sensitivity analysis on the neonatal hypoglycaemia outcome restricted to babies where testing has been performed as indicated by the British Association of Perinatal Medicine (BAPM) criteria [14] (i.e. excluding babies tested but not satisfying the BAPM criteria).

### Plans to give access to the full protocol, participant-level data, and statistical code {31c}

Requests for access to the trial protocol, anonymised trial dataset, and statistical codes will be considered by the chief investigator following the data-sharing policies of King’s College London and Birmingham Clinical Trials Unit, with input from the co-investigator group where applicable.

### Health economics analysis plan

A separate health economics analysis plan will be produced and will provide a more comprehensive description of the planned health economic analyses. A brief outline of these analyses is given below.

An economic evaluation will be conducted to determine the cost-effectiveness of nifedipine compared to labetalol from the perspective of the National Health Service (NHS). Our clinical hypothesis is that nifedipine will outperform labetalol in terms of reducing the incidence of severe systolic hypertension among pregnant women. Additionally, we hypothesise that the co-primary fetal/neonatal outcome will be non-inferior in the nifedipine group. Hence, we also anticipate no significant difference in costs for neonatal care between the two groups, but potential variations in costs and maternal health-related quality of life in favour of nifedipine due to better systolic blood pressure control throughout the trial period.

To demonstrate these hypotheses, we will collect data on healthcare resource utilisation for mothers during the trial period and for babies after delivery. For mothers, we will review case notes to gather information on antenatal care (including outpatient visits and hospital admissions prior to delivery), care received during delivery, and the duration of hospital stay post-delivery. Regarding babies, we will record the number of inpatient days categorised by the level of care required (high, medium, or low) after delivery. Unit costs for valuing the healthcare provided will be obtained from national references. We will assess maternal health-related quality of life at various time points using the EQ-5D-5L instrument: at trial entry, 2 weeks after randomisation, and every 4 weeks until the expected delivery date. The utility scores for EQ-5D-5L states will be derived from value sets currently being evaluated by the NICE [22], with the expectation of a final value set being available when the economic evaluation is analysed. To provide initial insights into the need for further extrapolation, we will initially present a cost-consequence analysis of the primary and secondary clinical outcomes, along with maternal quality of life and the costs associated with mother-baby pairs until the primary hospital discharge or 28 days post-birth.

Complications (e.g. pre-eclampsia) associated with hypertension during pregnancy are known to increase the risk of cardiovascular disease and affect long-term life expectancy, health-related quality of life, and costs [1]. Therefore, if the trial demonstrates that nifedipine can better control hypertension during pregnancy, there is potential to reduce associated complications and mitigate the long-term risks of cardiovascular disease, either for all women or specific sub-groups. In such a scenario, we will consider employing decision analytic modelling to examine costs and maternal quality-adjusted life years (QALYs) over a lifetime horizon, enabling a cost-utility analysis. This model will depict the disease progression of women with elevated blood pressure following delivery, incorporating cardiovascular events over time. Observed risk factors, quality of life estimates, and healthcare resource utilisation data from our trial will inform the characteristics of a hypothetical cohort for each drug therapy within the model. The model will be adapted from an existing economic model developed as part of the BUMP studies [23, 24]. To inform health states over time, we will extract information on maternal quality of life and costs from relevant literature sources. Costs and outcomes will be synthesised using an incremental cost-effectiveness ratio, expressed as the cost per QALY gained between the two therapies.

When interpreting and reporting this economic evaluation, we will adhere to current guidelines, giving special attention to addressing uncertainty in the within-trial and the decision analytical model [25, 26].

## Oversight and monitoring

### Composition of the coordinating centre and trial steering committee {5d}

The trial is coordinated by the Birmingham Clinical Trials Unit. All aspects of the conduct and progress of the trial and the day-to-day running of the trial are monitored by the trial management group (TMG). The TMG meets monthly and includes the chief investigator, senior statistician, senior trials manager*,* coordinating midwife, and other TMG members, with oversight from the clinical trials unit director as required. Each participating centre has a local principal investigator (PI) who will report to the TMG. The TMG reports to the trial steering committee (TSC). The TSC meets at least annually and as required depending on the needs of the trial and is responsible for providing overall oversight of the trial, including practical aspects of the trial and ensuring the trial is run safely for the participants in addition to providing appropriate data to the sponsor and funder. TSC members include an independent chair, three other independent members, a PPI representative, and the chief investigator. The TSC considers and acts, as appropriate, upon the recommendations of the DMC, and is responsible for deciding whether a trial needs to be stopped on grounds of safety or efficacy.

### Composition of the data monitoring committee, its role and reporting structure {21a}

The independent DMC was established for the sponsor to assess the progress of the trial and review safety data and critical efficacy endpoints and make recommendations to the sponsor on whether to continue or modify the trial.

Data analyses will be supplied in confidence to the independent DMC*,* who will be asked to advise on whether the accumulated data from the trial, together with the results from other relevant research, justify continuing recruitment of further women to the trial. The DMC meets at least annually as agreed by the DMC and documented in the DMC Charter unless there is a specific reason to amend the schedule*.* The DMC will report directly to the TSC who conveys the recommendations of the DMC to the funder.

### Adverse event reporting and harms {22}

Adverse events are collected for Giant PANDA study participants, and their babies, from consent (from birth for babies) up to primary discharge after birth or 28 days post-birth, whichever occurs sooner. The collection and reporting of adverse events are in accordance with the Medicines for Human Use Clinical Trials Regulations 2004 and its subsequent amendments. Adverse events are commonly encountered in this population of pregnant women as a part of the clinical condition of pregnancy and hypertension in pregnancy. As the safety profiles of labetalol and nifedipine are well-characterised, a strategy of targeted reporting of adverse events is used without affecting the safety of participants. Adverse events are separated into expected adverse events, which are recorded on the case note review but not reported, and those that are reportable.

Expected maternal (serious) adverse events:

- Admission in active labour
- Admission for cervical ripening or induction of labour
- Admission for caesarean section
- Admission for assessment for suspected fetal compromise, including poor growth, or reduced fetal movements
- Admission for monitoring for hypertension or pre-eclampsia, antepartum haemorrhage, suspected preterm labour, pre-labour rupture of the membranes or other reasons for monitoring
- Admission for psychiatric or social reasons
- Admission for unstable lie or external cephalic version
- Admission for postpartum complications
- Known complications of pregnancy and pregnancy hypertension that are collected for every woman as part of outcome collection

Expected fetal and neonatal (serious) adverse events:

Known fetal and neonatal complications of pregnancy that are collected for every infant as part of outcome collection include, but are not limited to:

- Neonatal unit admission
- Stillbirth after 24 weeks’ gestation
- Neonatal death up to 28 days
- Preterm delivery (< 37 completed weeks’ gestation)
- Neonatal complications (including but not limited to hypoglycaemia, seizures, encephalopathy, need for respiratory support, sepsis, intraventricular haemorrhage, confirmed infection, necrotising enterocolitis, retinopathy of prematurity, congenital anomaly, intraventricular haemorrhage)

Reportable serious adverse event:

- Maternal death
- Maternal stroke
- Stillbirth after 24 weeks’ gestation
- Neonatal death up to 28 days

Adverse events are recorded in the study database and assessed for severity, seriousness, and causality. Serious adverse events are required to be signed off by the site PI and CI and the coordinating centre ensures appropriate reporting to appropriate regulatory bodies in the appropriate timeframe. All reported serious adverse events are reviewed by the DMC.

### Frequency and plans for auditing trial conduct {23}

The central trial team monitors recruitment, provides study-related training, and monitors the quality of the data collected. The site research team is responsible for undertaking all trial-related activities at their recruiting site. The Giant PANDA study has been classed as a type A, low-risk, trial. This is comparable to the risk of standard medical care as both treatments are routinely used in clinical practice.

Given the low-risk nature of this study, central monitoring is routine, and onsite monitoring is triggered, or as required, as documented in the sponsor/quality management group-approved monitoring plan. The central trial team are in regular contact with the site research team to check on progress and address any queries. Trial staff regularly check the quality of the data collected for compliance with the protocol, data consistency, and completeness. The study data manager sends sites data clarification queries requesting missing data or clarification of inconsistencies or discrepancies. Additional on-site monitoring visits would be triggered, for example by poor electronic case report forms return. The PI must permit trial-related monitoring, audits, ethical review, and regulatory inspection(s) at their site, providing direct access to source data and documents.

### Plans for communicating important protocol amendments to relevant parties (e.g. trial participants, ethical committees) {25}

Protocol modifications would be communicated to the appropriate regulatory bodies (Research Ethics Committee (REC), the Medicines & Healthcare products Regulatory Agency (MHRA), and Health Research Authority (HRA)) and following approval communicated by the sponsor to each site (inclusive of the research management office and local research team). Monthly newsletters and site teleconferences provide a platform to communicate across sites, with discussions inclusive of recruitment progress and sharing challenges and good practices.

### Dissemination plans {31a}

Trial results will be communicated to healthcare providers and the scientific community via peer-reviewed journals, presentations at national and international conferences, and via the Royal College of Obstetricians and Gynaecologists. Furthermore, dissemination to the relevant community will be via pregnancy hypertension support groups and charities. Study findings will also be communicated to participants via a lay summary emailed to those who indicate they want this on their consent form.

## Discussion

As detailed above (in the ‘Trial design {8}’ section), the Giant PANDA study was initiated at the beginning of the COVID-19 pandemic. The trial design had already been streamlined for efficient delivery, including a pragmatic design, electronic case report forms, electronic participant completed questionnaires, electronic consent with a remote option, no additional study visits, usual clinical care prescriptions, and follow-up within clinical care, with a supportive central research team with trial-related training delivered online. These strategies were intended to reduce the burden to the site team; however, the practical issues of operating within the restrictions caused by the COVID-19 pandemic caused initial delays to the study timeline. Outside of pregnancy, prescribing decision are influenced by family origin or ethnicity across many hypertension guidelines. In order to address this, our analysis will seek to understand if tailoring blood pressure medication by ethnicity could improve outcomes for pregnant women and their babies, given the disproportionate burden and increased risk of adverse outcomes in women from ethnic minority backgrounds.

This study aims to fill the gap in the evidence and establish whether one drug or another is better for the woman (i.e. superior) and whether the outcomes for the infant are not worse (i.e. not inferior), adding to the sparse evidence on which women and clinicians share decision-making, with the trial results likely to influence clinical practice directly and via guideline changes.

## Trial status

The current version of the protocol is version 1.1, 11 January 2020. The trial received approval from MHRA on 16 December 2020 and HRA on 17 December 2020 (REC approval was granted on 3 November 2020). The trial opened to recruitment in June 2021, with the first participant recruited on 7 June 2021 and is estimated to complete recruitment by summer 2024.

### Supplementary Information

**Additional file 1.**

## Data Availability

Individuals with access to the full dataset will include the trial manager and data manager. The CI will have access to the full dataset after the database lock. The CI’s Institution will be the overall owner of the study data. Site investigators will not have access to the full data set and must not use, disseminate, or publish any trial data without the prior written consent of the CIG and TSC. Site-specific data will be provided to site PIs at the end of the study. In line with the conditions of use of the following validated questionnaires, Beliefs about Medicines Questionnaire (BMQ-Specific), Medication Adherence Report Scale (MARS-5), copyright holders will receive a copy of the de-identified study datasets relating only to these questionnaire responses. Requests for the dataset from appropriate academic parties will be considered by the CI following the data-sharing policies of King’s College London and the BCTU, with input from the co-investigator group where applicable.

## Abbreviations

BAPM: British Association of Perinatal Medicine
BMQ: Beliefs about Medicines Questionnaire
CI: Chief investigator
CI: Confidence interval
DMC: Data monitoring committee
EQ-5D-5L: EuroQol-5 Dimension
GCP: Good Clinical Practice
HRA: Health Research Authority
MARS: Medication Adherence Report Scale
MHRA: The Medicines & Healthcare products Regulatory Agency
NICE: National Institute for Health and Care Excellence
PI: Principal investigator
REC: Research Ethics Committee
RR: Risk ratio
TMG: Trial management group
TSC: Trial steering committee
TSQM: Treatment Satisfaction Questionnaire for Medication
