# Supplementary material for: A treatment strategy with nifedipine versus labetalol for women with pregnancy hypertension: study protocol for a randomised controlled trial (Giant PANDA)
Source: Trials. 2023 Sep 12;24:584. doi: 10.1186/s13063-023-07582-9 (PMC10496358; doi:10.1186/s13063-023-07582-9)
Supplement: Supplementary file 1 — Additional file 1. [file 13063_2023_7582_MOESM1_ESM.pdf]

UNIVERSITY OF BIRMINGHAM | BCTU Birmingham Clinical Trials Unit

Giant PANDA
Pregnancy ANtihypertensive Drugs: which Agent is best?

Trial Registration: ISRCTN

Statistical Analysis Plan

SAP Version Number	Protocol Version Number
1.0	1.0

Name of Author:	Catherine Hewitt	Role:	Trial Statistician	Affiliation:	BCTU University of Birmingham
Signature of Author:		Date:			
Name of Reviewer:	Pollyanna Hardy	Role:	Senior Statistician	Affiliation:	BCTU University of Birmingham
Signature of Reviewer:		Date:			
Name of Chief Investigator:	Lucy Chappell	Role:	Chief Investigator	Affiliation:	
Signature of Chief Investigator:		Date:			
**This Statistical Analysis Plan has been approved by:**					
Name of Approver:		Role:	Senior Statistician	Affiliation:	BCTU University of Birmingham
Signature of Approver:		Date:			

Statistical Analysis Plan (SAP) Amendments

SAP version number	SAP section number	Description of and reason for change	Timing of change with respect to interim analysis/ final analysis/ database lock	Blind Reviewer	
				Name:	
				Signature:	
				Date:	
				Name:	
				Signature:	
				Date:	
				Name:	
				Signature:	
				Date:	

Abbreviations & Definitions	
**Abbreviation / Acronym**	**Meaning**
APS	Antiphospholipid syndrome
BAPM	British Association of Perinatal Medicine
BCTU	Birmingham Clinical Trials Unit
BMI	Body Mass Index
BMQ	Beliefs about Medicine Questionnaire
CI	Confidence Interval
CONSORT	Consolidated Standards of Reporting Trials
DMC	Data Monitoring Committee
FiO_2	Fraction of inspired oxygen
ISRCTN	International Standard Randomised Controlled Trial Number
HDU	High Dependency Unit
HPC	Healthcare professional contact
IQR	Interquartile range
ITT	Intention to Treat
IUGR	Intrauterine growth restriction
KG	Kilograms
L	Litre
M	Metres
MARS-5	Medication Adherence Report Scale
MCMC	Markov chain Monte Carlo method
NICE	National Institute of Clinical Excellence
NICU	Neonatal Intensive Care Unit
NNU	Neonatal unit admission
SAE	Serious Adverse Event
SAP	Statistical Analysis Plan
SCBU	Special Care Baby Unit
SD	Standard Deviation
SGA	Small for gestational age
SLE	Systemic Lupus Erythematosus
SpO_2	Oxygen saturation
SUSAR	Suspected Unexpected Serious Adverse Reaction
TSC	Trial Steering Committee
**Term**	**Definition**
International Standard Randomised Controlled Trial Number	A clinical trial registry
Protocol	Document that details the rationale, objectives, design, methodology and statistical considerations of the study
Randomisation	The process of assigning trial subjects to intervention or control groups using an element of chance to determine the assignments in order to reduce bias.
Statistical Analysis Plan	Pre-specified statistical methodology documented for the trial, either in the protocol or in a separate document.

TABLE OF CONTENTS

1. Introduction ... 6
2. Background and rationale ... 6
3. Trial objectives ... 6
4. Trial methods ... 6
4.1. Trial design ... 6
4.2. Trial interventions ... 6
4.3. Primary outcome measure ... 7
4.4. Secondary outcome measures ... 7
4.5. Timing of outcome assessments ... 9
4.6. Randomisation ... 9
4.7. Sample size ... 10
4.8. Framework ... 10
4.9. Interim analyses and stopping guidance ... 10
4.10. Internal Pilot Progression Rules ... 11
4.11. Timing of final analysis ... 11
4.12. Timing of other analyses ... 12
4.13. Trial comparisons ... 12
5. Statistical Principles ... 12
5.1. Confidence intervals and p-values ... 12
5.2. Adjustments for multiplicity ... 12
5.3. Analysis populations ... 12
5.4. Definition of adherence ... 12
5.5. Handing protocol deviations ... 13
5.6. Unblinding ... 13
6. Trial population ... 13
6.1. Recruitment ... 13
6.2. Baseline characteristics ... 13
7. Interventions ... 14
7.1. Description of the interventions ... 14
7.2. Adherence to allocated intervention ... 14
8. Protocol deviations ... 14
9. Analysis methods ... 14
9.1. Covariate adjustment ... 14
9.2. Distributional assumptions and outlying responses ... 14
9.3. Handling missing data ... 14
9.4. Data manipulations ... 15
9.5. Analysis methods – co-primary outcomes ... 15
9.6. Analysis methods – secondary outcomes ... 15
9.7. Analysis methods – exploratory outcomes and analyses ... 16
9.8. Safety data ... 16
9.9. Planned subgroup analyses ... 16
9.10. Sensitivity analyses ... 16
10. Analysis of sub-randomisations ... 17
11. Health economic analysis ... 17
12. Statistical software ... 17
13. References ... 17
Appendix A: Deviations from SAP ... 20
Appendix B: Trial schema ... 21
Appendix C: Schedule of assessments ... 22
Appendix D: Template report ... 23

Appendix E: Data manipulations 24
Appendix F: Observational study 36

1. Introduction

This document is the Statistical Analysis Plan (SAP) for the Giant PANDA trial, and should be read in conjunction with the current trial protocol. This SAP details the proposed analyses and presentation of the data for reporting the results of the Giant PANDA trial.

Analysis methods will follow the strategy set out here. Subsequent analyses of a more exploratory nature will not be bound by this strategy, though they are expected to follow the broad principles laid down here. The principles are not intended to curtail exploratory analysis (e.g. to decide cut-points for categorisation of continuous variables), nor to prohibit accepted practices (e.g. transformation of data prior to analysis), but they are intended to establish rules that will be followed, as closely as possible, when analysing and reporting data.

Any deviations from this SAP will be described and justified in the final report or publication of the trial (using a table as shown in Appendix A). The analysis will be carried out by an appropriately qualified statistician, who should ensure integrity of the data during their data cleaning processes.

2. Background and rationale

The background and rationale for the trial are outlined in detail in the protocol. In brief, antihypertensive drug choice in pregnancy is currently largely arbitrary despite The National Institute for Health and Care Excellence (NICE) guidelines detailing labetalol as first line treatment based on its licensed status. This study aims to fill the gap in the evidence and enable evaluation of maternal and infant benefits and risks for antihypertensive prescribing (labetalol versus nifedipine) in pregnancy. Giant PANDA is a two-arm randomised controlled trial, to establish whether one drug is better for the woman (i.e. superior) and whether the outcomes for the infant are not worse (i.e. not inferior) and add to the sparse evidence on which women and clinicians share value-based decision-making.

3. Trial objectives

The primary objective is to evaluate if treatment with nifedipine (calcium channel blocker), compared to labetalol (mixed alpha/beta blocker) in women with pregnancy hypertension, reduces severe maternal hypertension without increasing fetal or neonatal death, or neonatal unit admission.

Secondary objectives are as follows:

- To investigate the effect of treatment with nifedipine versus labetalol on other secondary maternal and fetal/neonatal outcomes including patient-reported outcome measures.
- To evaluate the cost-effectiveness of nifedipine versus labetalol as antihypertensive drugs from an NHS perspective.

4. Trial methods

4.1. Trial design

Giant PANDA is a prospective, late phase, pragmatic, parallel group, open-label, multicentre, two-arm randomised controlled trial. Women who decline randomisation or are unable to be randomised (due to contraindications to either labetalol or nifedipine or women taking both drugs and not able to be randomised to a single drug) will be offered participation in an observational study, involving data collection only (as detailed in Appendix F).

Participants will be recruited from secondary care. Giant PANDA includes an internal pilot phase with progression rules (refer to section 4.10).

4.2. Trial interventions

Intervention group: Oral nifedipine modified release preparations (no brand specified) to be taken

twice daily. The starting dose will be left to the discretion of the responsible healthcare professional, guided by blood pressure on the day, previous antihypertensive dose (where applicable) and any other relevant factors. The starting dose is usually nifedipine-modified release 10mg twice a day, increased to a maximum of 40mg twice daily.

Active control group: Oral labetalol (no brand specified) to be taken twice to three times daily. The starting dose will be left to the discretion of the responsible healthcare professional, guided by blood pressure on the day, previous antihypertensive dose (where applicable) and any other relevant factors. The starting dose is usually labetalol 100mg twice a day, increased to a maximum of 2,400mg total daily dose, divided into three or four times a day regimen.

4.3. Primary outcome measure

Maternal: Severe hypertension (proportion of days with a healthcare professional measured systolic blood pressure reading ≥160mmHg between randomisation and birth). For outpatient visits: the highest systolic blood pressure reading at each visit will be recorded; for inpatient admissions, the highest systolic blood pressure reading per day will be recorded inclusive of the day of birth (up to the time of birth). All included blood pressure readings will be measured by healthcare professionals.

Fetal/neonatal: Composite of fetal loss before birth or known neonatal death, or neonatal unit admission involving separation of the baby from the mother between randomisation up to primary hospital discharge or 28 days post-birth, whichever occurs sooner (with no double counting of outcomes).

Please refer to Appendix E (data manipulations) for the details of how the primary outcome will be derived.

4.4. Secondary outcome measures

For the purposes of assessing the effect of the randomised allocation to labetalol or nifedipine within the trial, outcomes will be collected from randomisation up to primary hospital discharge for each of the woman or baby post-birth, or 28 days post-birth if remains in hospital, whichever occurs sooner.

[For the observational study outcomes will be collected from consent up to primary hospital discharge for each of the woman or baby post-birth, or 28 days post-birth, whichever occurs sooner.]

All safety data (adverse events and serious adverse events) will be collected from consent. Outcomes have been chosen to align with those from the Core Outcome Set for Pre-eclampsia wherever possible [1].

Outcomes indicated by an asterisk (*) will be presented with a treatment effect and confidence intervals. All other outcomes will be presented with summary statistics only.

The secondary outcomes are as follows:

Maternal:

Up to birth:

- Severe maternal hypertension* (defined as any episode of severe maternal hypertension (systolic blood pressure ≥160 mmHg between randomisation and birth)) [2]
- Mean antenatal systolic blood pressure* (using highest systolic blood pressure per day as collected for the primary outcome)

- Mean antenatal diastolic blood pressure (using highest diastolic blood pressure per day)
- Proportion of days with an antenatal systolic hypertension blood pressure reading ≥140mmHg
- Proportion of days with an antenatal diastolic hypertension blood pressure reading ≥90mmHg
- New diagnosis of pre-eclampsia*
- Diagnosis of eclampsia
- Diagnosis of Haemolysis, Elevated Liver enzymes, Low Platelets syndrome (HELLP)
- Placental abruption
- Severe maternal morbidity* (fullPIERS consensus definition [3])
    - Components of severe maternal morbidity (as defined above)
- Maternal death
- Maternal stroke
- Prescription of additional antihypertensive drug(s)
- Prescription of alternative antihypertensive drug(s)
- Persistence with allocated antihypertensive (time from randomisation to first discontinuation)
- Discontinued allocated antihypertensive drug*
- Undesirable effects of allocated (and other) antihypertensive drug(s) (number of women* and number of undesirable effects)
- Total number of antenatal hospital inpatient days.

Medication-related self-reported outcomes (measured at 2 weeks post-randomisation, if prior to birth) using validated tools:
- Treatment satisfaction with allocated antihypertensive drug [4]
- Beliefs about allocated antihypertensive drug [5]
- Adherence to allocated (and other) antihypertensive drug(s) [6]

At delivery/birth:
- Indicated delivery* (induction of labour or prelabour rupture of membranes (PROM) with stimulation of labour or pre-labour Caesarean section)
- Mode of onset of birth (spontaneous, induction of labour, PROM with stimulation of labour, pre-labour Caesarean section)
    - Indication for onset of birth

Between birth and primary hospital discharge or 28 days post-birth, whichever occurs sooner:
- New episodes of severe maternal morbidity (fullPIERS consensus definition [3])
    - Components of severe maternal morbidity (as defined above)
- Maternal death

Fetal and neonatal:

Between birth and primary hospital discharge or 28 days post-birth, whichever occurs sooner, using denominator of all fetuses/infants:
- Fetal loss prior to 24 weeks' gestation
- Fetal loss ≥24+0 weeks' gestation (stillbirth)
- Known early neonatal death (up to 7 days from birth)
- Known late neonatal death (between 7 and up to 28 days from birth)
- Neonatal unit admission* (separation of baby from mother)
- Principal recorded indication for neonatal unit admission

- Length of stay in neonatal unit (and level of care)
- Major congenital abnormality as defined by EUROCAT*
- Mode of birth (spontaneous vaginal*, assisted vaginal, Caesarean section)
- Indication for mode of birth
- Gestational age at birth*
- Preterm birth (<37 completed weeks' gestation)
- Preterm birth (<32 completed weeks' gestation)
- Birthweight
- Birthweight centile*
- Birthweight small for gestational age (<10th centile for gestational age)
- Umbilical arterial pH <7 at birth
- Apgar score at 5 mins after delivery
- Need for additional resuscitation at birth: intubation in the delivery room, resuscitation drugs or chest compressions
- Need for respiratory support
  - Type of respiratory support needed
- Need for treatment for neonatal hypoglycaemia* (in those having blood glucose monitoring)
  - Type of treatment for hypoglycaemia
- Lowest blood glucose measurement within the first 48 hours after birth
- Neonatal seizures
- Intracranial haemorrhage
- Necrotising enterocolitis

Process outcomes:

- Number of babies in whom blood glucose monitoring was indicated at birth
- Indication for blood glucose monitoring
- Blood glucose test performed

Adverse events:

- Adverse event recorded (number of women and number of adverse events)
- Adverse event recorded (number of fetuses/neonates and number of adverse events)

Please refer to Appendix E (data manipulations) for the details of how the secondary outcomes will be derived.

4.5. Timing of outcome assessments

The schedule of trial procedures and outcome assessments are given in Appendix C.

4.6. Randomisation

Participants will be randomised in a 1:1 ratio to either nifedipine or labetalol.

Randomisation will be provided by a secure online randomisation system at the Birmingham Clinical Trials Unit (BCTU). A minimisation algorithm will be used within the online randomisation system to ensure balance in the treatment allocation over the following variables:

- Maternity unit
- Hypertension type (chronic, gestational, pre-eclampsia)

- Diabetes (yes, no)
- Singleton (yes, no)
- Self-reported ethnicity (black, all other)
- Gestational age (11+0 to 19+6, 20+0 to 27+6, 28+0 to 34+6 weeks' gestation).

A 'random element' will be included in the minimisation algorithm. Full details of the randomisation specification will be stored in a confidential document at BCTU.

4.7. Sample size

The sample size calculations are driven by the fetal/neonatal co-primary outcome. Assuming a control group event rate for fetal or neonatal death or neonatal unit admission of 25% [7, 8], to test a non-inferiority hypothesis on this fetal/neonatal outcome, a total sample size of 2,190 babies will have 90% power to detect a non-inferiority margin of 6%, with a 2.5% one-sided significance level. This would provide robust, clinically meaningful evidence to assess the impact of women taking nifedipine on neonatal outcomes compared to those taking labetalol. Based on ONS birth statistics, the proportion of women in the target population expected to have multi-fetal pregnancies is around 1.5%, but the proportion recruited to this trial is uncertain. A sample size of 2,190 women will provide a conservative estimate for the number of babies required to address the hypothesis for the fetal/neonatal co-primary outcome. Due to the anticipated small proportion of multi-fetal pregnancies, this sample size will also allow for the dependence between outcomes for infants from the same pregnancy [9].

For the maternal co-primary outcome, using the dataset from the PANDA feasibility study [8] (112 pregnant women with chronic hypertension), the mean proportion of days with clinic and hospital blood pressure measurements ≥160mmHg was 9.6% with a standard deviation of 16.4%. The inclusion of women with gestational hypertension in the Giant PANDA study is not expected to impact on these estimates substantially. A sample size of 2,190 (which allows for a 6% margin of non-inferiority for the neonatal outcome), will mean that we can detect a 2.3% superiority difference between the mean proportions, equivalent to an effect size of 0.14 of a standard deviation, based on a two sample t-test (5% two-sided alpha, 90% power), e.g. from around a mean of 9.6% to 11.9%. Although the data are expected to be highly skewed, the approximation to the normal distribution has been shown to produce conservative estimates of the sample size [10]. With this sample size, a clinically meaningful non-inferiority margin for the fetal/neonatal co-primary outcome can be detected whilst allowing a feasible trial to be conducted (a non-inferiority margin of 5% would require 3,160 women). This sample size also retains power to detect a 5.5% reduction in severe hypertension (from 22% to 16.5%) measured as a binary secondary outcome.

Allowing for up to 5% loss to follow-up, as in similar trials [8, 11] would require a total sample size of approximately 2,300 women, 1150 women per group.

4.8. Framework

The objective of the trial is to test both the superiority of nifedipine compared with labetalol for severe maternal hypertension and the non-inferiority of nifedipine compared with labetalol for neonatal/fetal death or NNU admission.

The null hypothesis for the maternal co-primary outcome is that there is no difference in severe maternal hypertension between the intervention groups. The null hypothesis for the fetal/neonatal co-primary outcome is that there is a difference in the composite outcome of fetal loss, neonatal death or NNU admission between the intervention groups.

4.9. Interim analyses and stopping guidance

A separate Data Monitoring Committee (DMC) reporting template will be drafted and agreed by the

DMC including an agreement on which outcomes will be reported at interim analyses. The statistical methods stated in this SAP will be followed for the outcomes included in the DMC report, where possible.

Interim analyses of safety and efficacy outcomes will take place during the study. Formal statistical methods will be used as guidelines rather than absolute rules with respect to the possible recommendations listed above. This is because they generally only consider one dimension of the trial. Reasons for disregarding stopping guidelines should be recorded. The guideline in the Giant PANDA trial is that DMC should consider whether the randomised comparisons in the trial have provided both (a) "proof beyond reasonable doubt" that for all, or some, types of patient either policy is definitely indicated or definitely contraindicated in terms of a net difference in the major endpoints, and (b) evidence that might reasonably be expected to influence the patient management of many clinicians who are already aware of the other main trial results. "Proof beyond reasonable doubt" may be considered a difference equivalent to $p<0.001$ in the analysis of a major end-point needed to justify halting the study prematurely. If this criterion were to be adopted then there would be minimal concern regarding the inflation of type-I error rates for the final analysis regardless of how many interim analysis were performed (within reason). This guideline is broadly similar to the Haybittle-Peto approach.

4.10. Internal Pilot Progression Rules

An embedded internal pilot will run in 17 units (staggered start) over a period of ten months to assess recruitment and retention rates, acceptability and implementation. Pre-specified progression criteria have been agreed as follows:

	**Black (<67% of target)**	**Red (67-84% of target)**	**Amber (85% of target)**	**Green (actual target)**
Number of sites open	≤11	12-14	15-16	17
Recruitment (per centre/month)*	<2.00	2.00-2.54	2.55-2.99	3.00
Cumulative recruitment target	<195	195-246	247-290	291
Actions	Discuss with TSC and consider stopping trial	Discuss with TSC strategies for improvement and consider changes to processes including opening further sites	Continue, with review of strategies to improve at existing sites	Continue

*Excluding two month lag phase in each centre.

In the light of the ongoing uncertainties during the COVID-19 pandemic and ongoing disruption to maternity care and Research and Development Services, additional actions (e.g. increasing number of sites opened and/or reviewing initiation of intervention delivery through remote means) to support recruitment may be necessary to achieve the pilot targets.

4.11. Timing of final analysis

The final analysis for the trial will occur after all women and neonates have been discharged or reached 28-days post-birth (which ever comes sooner) and the corresponding outcome data has been entered onto the trial database and validated as being ready for analysis. This is provided that the trial has not been stopped early for any reason (e.g. DMC advice or funding body request).

4.12. Timing of other analyses

Not applicable.

4.13. Trial comparisons

All references to 'group' refer to nifedipine or labetalol, for the analysis of all women randomised. See Appendix F for definitions relating to the observational cohort.

5. Statistical Principles

5.1. Confidence intervals and p-values

All estimates of differences between groups will be presented with two-sided 95% confidence intervals, unless otherwise stated. A p-value will be reported from a two-sided test at the 5% significance level for the maternal co-primary outcome measure only. No p-value will be reported for the fetal/neonatal co-primary outcome measure, since this is a non-inferiority outcome and we are only interested in the upper limit of the 95% confidence interval for inferring if Nifedipine is 'non-inferior' to Labetalol.

5.2. Adjustments for multiplicity

No correction for multiple testing will be made.

5.3. Analysis populations

All primary analyses (primary and secondary outcomes including safety outcomes) will be by intention-to-treat (ITT) [12]. Participants will be analysed in the intervention group to which they were randomised, and all participants shall be included whether or not they received the allocated intervention. The ITT analysis will ensure a comparison that maintains the rigour of randomisation but could risk providing results that are biased towards non-inferiority for the fetal/neonatal co-primary outcome. Per-protocol analyses will also be carried out as sensitivity analyses for the two co-primary-outcome measures. Refer to section 5.4 for definitions of adherence and definitions of the per protocol groups. Refer to section 9.10 for further details on sensitivity analyses.

5.4. Definition of adherence

Adherence to the randomised allocated intervention will be monitored by prescriptions and self-reported adherence. Prescription data are collected at antenatal inpatient contacts, antenatal outpatient contacts, each study contact (at two weeks, then at six weeks and every 4 weeks after that) and birth. Self-reported adherence data is collected at the two week study contact then at the six week study contact and every 4 weeks after that. Adherence will be described as short-term and longer-term according to the following criteria:

Short-term adherence

(i). Prescribing data: At each assessment where prescription data is collected up to two weeks post-randomisation or birth whichever occurs first, if all prescriptions include their randomised allocated antihypertensive drug (even if additional antihypertensive drugs are prescribed).

(ii). Self-reported adherence data: ≥90% self-reported adherence at the two week study contact.

Short-term adherence to the randomised allocated intervention will be classified for each woman using two definitions:

a) Criterion (i) is met (regardless if criterion (ii) is met).

b) Criteria (i) and (ii) are met.

Two per-protocol populations for short-term adherence will be defined as those women who meet each of (a) and (b) above.

Longer-term adherence

(iii). Prescription data: At each assessment where prescription data is collected, if all prescriptions include their randomised allocated antihypertensive drug (even if additional antihypertensive drugs are prescribed).

(iv). Self-reported adherence data: ≥90% self-reported adherence across all study contact(s). Where data on multiple study contacts are collected a time-adjusted average of self-reported adherence will be used. For example, if a woman reports taking their study drug sometimes (50%) at the two week contact (asked for the previous two weeks), always (100%) at the six week contact (asked for the previous four weeks), and often (90%) at the ten week contact (asked for the previous four weeks), the time-adjusted self-reported adherence will be calculated as:

[(50*2)+(100*4)+(90*4)]/10=86%

Longer-term adherence to the randomised allocated intervention will be classified for each woman using two definitions:

c) Criterion (iii) is met (regardless if criterion (iv) is met).
d) Criteria (iii) and (iv) are met.

Two per-protocol populations for longer-term adherence will be defined as those women who meet each of (c) and (d) above.

Women who deliver prior to the two-week contact (i.e. no data on self-reported adherence)
For both short-term and longer-term definitions of adherence, in women who deliver prior to the two-week contact (i.e. no data on self-reported adherence), if criterion (i) (short-term adherence) or criterion (iii) (longer-term adherence) are satisfied, they will be regarded as adherent. If criterion (i) (short-term adherence) or criterion (iii) (longer-term adherence) are not satisfied, they will be regarded as non-adherent.

5.5. Handing protocol deviations

A protocol deviation is defined as a failure to adhere to the protocol such as errors in applying the inclusion/exclusion criteria, the incorrect intervention being given, incorrect data being collected or measured, follow-up visits outside the visit window or missed follow-up visits. We will apply a strict definition of the ITT principle and will include all participants as per the ITT population described in section 5.3 in the analysis, in some form, regardless of deviation from the protocol [12]. This does not include those participants who have specifically withdrawn consent for the use of their data in the first instance; however these outcomes will be explored as per other missing responses.

5.6. Unblinding

Not applicable, Giant PANDA is an open-label study.

6. Trial population

6.1. Recruitment

A flow diagram (as recommended by CONSORT [13]) will be produced to describe the participant flow through each stage of the trial. This will include information on the number (with reasons) of losses to follow-up (drop-outs and withdrawals) over the course of the trial. A template for reporting this is given in section 3.1 of the supplementary template report.

6.2. Baseline characteristics

The trial population will be tabulated as per section 3.4 of the supplementary template report. Categorical data will be summarised by number of participants, counts and percentages. Continuous data will be summarised by the number of participants, mean and standard deviation if deemed to be

normally distributed or number of participants, median and interquartile range if data are skewed, and ranges if appropriate. Tests of statistical significance will not be undertaken, nor confidence intervals presented [14].

7. Interventions

7.1. Description of the interventions

A template for reporting information on the interventions is given in sections 3.5 and 3.7 of the supplementary template report.

7.2. Adherence to allocated intervention

A cross-tabulation of allocated intervention by the adherence categories stated in section 5.4 will be produced (proportions and percentages). A template for reporting adherence is given in section 3.5 of the supplementary template report.

8. Protocol deviations

Frequencies and percentages by group will be tabulated for the protocol deviations as per section 3.3 of the supplementary template report.

9. Analysis methods

Intervention groups will be compared using regression models, or a similar method, to adjust for all covariates as specified in section 9.1, where possible.

9.1. Covariate adjustment

In the first instance, intervention effects between groups for all outcomes will be adjusted for the minimisation parameters listed in section 4.6. Categorised continuous variables (gestational age) will be treated as a continuous variable in this adjustment. Maternity unit will be treated as a random effect in the model (where possible), and all other factors as fixed effects. The co-primary maternal outcome and mean antenatal systolic blood pressure (secondary outcome) will also be adjusted for baseline systolic blood pressure (taken as the highest systolic blood pressure at the most recent HCP contact).

If covariate adjustment is not possible (e.g. the model does not converge), maternity unit will be removed first. If this reduced model still fails to converge, unadjusted estimates will be produced and it will be made clear in the final report why this occurred (e.g. not possible due to low event rate/lack of model convergence).

For binary outcomes only, if the (full) adjusted log-binomial model fails to converge, a Poisson regression model with robust standard errors will be used to estimate the same parameters [15]. If this also fails to converge, estimates will be produced from the log-binomial model (following rules for removal of variables as outlined above). It will be made clear in the final report why this occurred (e.g. not possible due to low event rate/lack of model convergence).

9.2. Distributional assumptions and outlying responses

Distributional assumptions (e.g. normality for continuous outcomes) will be assessed visually prior to analysis or tabulation. If responses are considered to be particularly skewed medians and interquartile ranges will be presented as opposed to means and standard deviations and non-parametric analysis techniques will be implemented.

9.3. Handling missing data

In the first instance, analysis will be completed on received data only with every effort made to follow-up participants to minimise any potential for bias. To examine the possible impact of missing data on the results, and to make sure we are complying with the intention-to-treat principle, sensitivity analysis will be performed on the co-primary outcome measures [16]. See section 9.10 for further details regarding sensitivity analyses.

9.4. Data manipulations

See Appendix E.

9.5. Analysis methods – co-primary outcomes

A template for reporting the co-primary outcome measures is given in section 3.6 of the supplementary template report.

The maternal co-primary outcome measure will be summarised using means and standard deviations. A generalised linear model will be used with a link function that provides the best fit for the data to calculate the mean difference in proportions (and 95% confidence interval) with a corresponding p-value.

The fetal/neonatal co-primary outcome measure will be summarised using frequencies and percentages. A log-binomial model will be used to generate an adjusted relative risk (and 95% confidence interval). Adjusted risk differences (and 95% confidence interval) will also be presented (using an identity link function).

See section 9.1 for covariate adjustment and model convergence.

9.6. Analysis methods – secondary outcomes

A template for reporting the secondary outcomes is given in section 3.7 of the supplementary template report. See section 9.1 for covariate adjustment and model convergence.

For continuous secondary outcome measures (mean antenatal systolic blood pressure, birthweight centile, gestational age at birth) which are normally distributed, means and standard deviations will be reported alongside adjusted mean differences (with 95% confidence intervals) estimated using a linear regression model. For continuous secondary outcome measures which are non-normally distributed, medians and interquartile ranges will be reported alongside unadjusted differences in medians using bootstrapping methods (repetition=1000, seed=123456).

Binary secondary outcome measures (new diagnosis of pre-eclampsia, severe maternal morbidity, discontinued allocated antihypertensive drug, indicated delivery, neonatal unit admission, major congenital abnormality, spontaneous vaginal mode of birth and treatment for hypoglycaemia) will be summarised using frequencies and percentages. A log-binomial model will be used to generate an adjusted relative risk (and 95% confidence interval). An adjusted risk difference (and 95% confidence interval) will also be presented.

Undesirable effects of allocated antihypertensive drug(s) will be summarised using frequencies and percentages. For side effects (excluding other) which occur with a frequency of >5% in at least one of the treatment groups a log-binomial model will be used to generate an adjusted relative risk (and 95% confidence interval). Adjusted risk differences (and 95% confidence interval) will also be presented.

All other secondary outcomes will be summarised using descriptive statistics only. Categorical data will be summarised by frequencies and percentages. Continuous data will be summarised by the number of responses, mean and standard deviation if deemed to be normally distributed and number of responses, median and interquartile range if data appear skewed. Time to event outcomes (Persistence with allocated antihypertensive [time from randomisation to first discontinuation]) will be summarised using medians and interquartile ranges. A Kaplan Meier plot will be produced to assess the data visually. Formal statistical testing will not be applied.

9.7. Analysis methods – exploratory outcomes and analyses

Any trial data that does not form a pre-specified outcome will be presented using simple summary statistics by intervention group (i.e. numbers and percentages for binary data and means (or medians) and standard deviations (or inter-quartile ranges) for continuous normal (or non-normal) data.

Further exploratory analyses will include an examination of blood pressure outcomes at 2 weeks post-randomisation. These include severe maternal hypertension (as per the primary outcome), severe maternal hypertension (yes/no), mean antenatal systolic blood pressure and mean antenatal diastolic blood pressure.

Please see Appendix F for details of how data from the observational cohort will be analysed.

9.8. Safety data

The number and percentage of women and fetuses/neonates experiencing any serious adverse events (SAEs) and suspected unexpected serious adverse reactions (SUSARs) will be presented by intervention group alongside the number of events reported. A log-binomial model will be used to generate an adjusted relative risk (and 95% confidence interval). An adjusted risk difference (and 95% confidence interval) will also be presented. See section 9.1 for covariate adjustment and model convergence.
A template for reporting this safety data is given in section 3.8 of the supplementary template report.

9.9. Planned subgroup analyses

Interpretation of subgroup analyses will be treated with caution (output will be treated as exploratory rather than definitive [17]). Analyses will be limited to the co-primary outcomes only, and the following subgroups:

- Hypertension type (chronic, gestational, pre-eclampsia)
- Diabetes (yes, no)
- Singleton (yes, no)
- Self-reported ethnicity (black, all other)
- Gestational age (11+0 to 19+6, 20+0 to 27+6, 28+0 to 34+6 weeks' gestation).

The effects of these subgroups will be examined by including a treatment group by subgroup interaction parameter in the regression models. P-values from tests for statistical heterogeneity will be presented alongside the effect estimate and 95% confidence intervals within subgroups. A template for reporting the subgroup analyses for the co-primary outcomes is given in section 3.6.4 of the supplementary template report.

9.10. Sensitivity analyses

Sensitivity analyses will be limited to the co-primary outcomes only and the secondary outcome assessing neonatal hypoglycaemia, and will consist of:

Sensitivity analyses on both co-primary outcomes

- Per-protocol analyses using short-term definitions of adherence (restricted to the populations described in sections 5.3 and 5.4).
- Per-protocol analyses using longer-term definitions of adherence (restricted to the populations described in sections 5.3 and 5.4).

Sensitivity analyses on the maternal co-primary outcome only

- To explore the influence, if any, of blood pressure measurement setting on the maternal co-primary outcome, an additional sensitivity analyses will include an analysis where the primary

outcome is derived using data from all blood pressure readings (in clinic and self-measured, reported in a telephone consultation).

- A restricted analysis including only women who self-monitor their blood pressure (defined as woman who report at the two week contact self-measuring their blood pressure 4-5 times or more in the last week).
- An analysis to assess the effect of missing responses via a multiple imputation approach. Maternal outcome in the nifedipine group and the labetalol group will be imputed separately, which would allow unbiased estimates for any interaction effects between the treatment and covariates in the analysis model. Missing responses will be simulated using a Markov chain Monte Carlo method (MCMC) that assumes an arbitrary missing data pattern and a multivariate normal distribution. Variables including the minimisation variables (with the exception of maternity unit) and baseline systolic blood pressure will be included in the model and used to generate 20 simulated data sets. Analysis will be then be performed (using the same methods described in section 9.5) on each set with the results combined using Rubin's rule to obtain a single set of results (treatment effect estimate and confidence intervals).

Sensitivity analyses on the fetal/neonatal co-primary outcome only

- An analysis to assess the effect of missing responses where any participants with missing outcome data in the nifedipine group will be treated as meeting the outcome (composite of fetal/neonatal loss, neonatal death up to 7 days) and missing outcome data in the labetalol group will be treated as not meeting the outcome.
- An analysis to assess the effect of missing responses where any participants with missing outcome data in the nifedipine group will be treated as not meeting the outcome and missing outcome data in the labetalol group will be treated as meeting the outcome (composite of fetal/neonatal loss, neonatal death up to 7 days).

Sensitivity analysis on the neonatal hypoglycaemia outcome

- Since there is a risk of measurement bias for the secondary outcome assessing neonatal hypoglycaemia, we will perform a sensitivity analysis restricted to babies where testing has been performed as indicated by the BAPM criteria (British Association of Perinatal Medicine 2017 [18]) (i.e. excluding babies tested but not satisfying the BAPM criteria).

10. Analysis of sub-randomisations

Not applicable.

11. Health economic analysis

As indicated in the protocol there will also be an economic analysis. The details of this analysis are documented separately.

12. Statistical software

Statistical analysis will be undertaken in the following statistical software packages: SAS software and Stata.

13. References

1. Duffy, J. M. N., A. E. Cairns, D. Richards-Doran, J. van 't Hooft, C. Gale, M. Brown, L. C. Chappell, W. A. Grobman, R. Fitzpatrick, S. A. Karumanchi, A. Khalil, D. N. Lucas, L. A. Magee, B. W. Mol, M. Stark, S. Thangaratinam, M. J. Wilson, P. von Dadelszen, P. R. Williamson, S. Ziebland and R. J. McManus (2020). "A core outcome set for pre-eclampsia research: An international consensus development study." BJOG: An International Journal of Obstetrics & Gynaecology.
2. Edgardo Abalos, Lelia Duley, D Wilhelm Steyn, Celina Gialdini, Antihypertensive drug therapy for mild to moderate hypertension during pregnancy, Cochrane database of systematic reviews,

2018, https://doi.org/10.1002/14651858.CD002252.pub4.
3. Von Dadelszen, P., B. Payne, J. Li, J. M. Ansermino, F. Broughton Pipkin, A. M. Cote, M. J. Douglas, A. Gruslin, J. A. Hutcheon, K. S. Joseph, P. M. Kyle, T. Lee, P. Loughna, J. M. Menzies, M. Merialdi, A. L. Millman, M. P. Moore, J. M. Moutquin, A. B. Ouellet, G. N. Smith, J. J. Walker, K. R. Walley, B. N. Walters, M. Widmer, S. K. Lee, J. A. Russell and L. A. Magee (2011). "Prediction of adverse maternal outcomes in pre-eclampsia: development and validation of the fullPIERS model." Lancet 377(9761): 219-227
4. https://www.iqvia.com/landing/treatment-satisfaction-questionnaire-for-medication-tsqm
5. Horne R, Weinman J, Hankins M. The beliefs about medicines questionnaire: the development and evaluation of a new method for assessing the cognitive representation of medication. Psychol Health. 1999;14(1):1–24
6. Chan AH, Horne R, Hankins M, Chisari C. The Medication Adherence Report Scale: A measurement tool for eliciting patients' reports of nonadherence. British Journal of Clinical Pharmacology. 2020 May 18
7. Magee, L. A., P. von Dadelszen, E. Rey, S. Ross, E. Asztalos, K. E. Murphy, J. Menzies, J. Sanchez, J. Singer, A. Gafni, A. Gruslin, M. Helewa, E. Hutton, S. K. Lee, T. Lee, A. G. Logan, W. Ganzevoort, R. Welch, J. G. Thornton and J. M. Moutquin (2015). "Less-tight versus tight control of hypertension in pregnancy." The New England Journal of Medicine 372(5): 407-417
8. Webster, L. M., J. E. Myers, C. Nelson-Piercy, K. Harding, J. K. Cruickshank, I. Watt-Coote, A. Khalil, C. Wiesender, P. T. Seed and L. C. Chappell (2017). "Labetalol Versus Nifedipine as Antihypertensive Treatment for Chronic Hypertension in Pregnancy: A Randomized Controlled Trial." Hypertension.
9. Yelland, L. N., T. R. Sullivan, C. T. Collins, D. J. Price, A. J. McPhee and K. J. Lee (2018). "Accounting for twin births in sample size calculations for randomised trials." Paediatr Perinat Epidemiol 32(4): 380-387.
10. Cundill, B. and N. D. Alexander (2015). "Sample size calculations for skewed distributions." BMC Medical Research Methodology 15: 28.
11. Duhig, K. E., J. Myers, P. T. Seed, J. Sparkes, J. Lowe, R. M. Hunter, A. H. Shennan, L. C. Chappell and P. t. group (2019). "Placental growth factor testing to assess women with suspected pre-eclampsia: a multicentre, pragmatic, stepped-wedge cluster-randomised controlled trial." Lancet 393(10183): 1807-1818.
12. Gupta SK. Intention-to-treat concept: A review. Perspect Clin Res. 2011;2(3):109-112.
13. Schulz KF, Altman DG, Moher D, for the CONSORT Group. CONSORT 2010 Statement: updated guidelines for reporting parallel group randomised trials. BMJ. 2010;340:c332.
14. Altman DG, Dore CJ. Randomisation and baseline comparisons in clinical trials. Lancet. 1990;335:149–53.
15. Zou G. A modified Poisson regression approach to prospective studies with binary data. Am J Epidemiol. 2004;159(7):702-6.
16. White IR, Horton NJ, Carpenter J, Pocock SJ. Strategy for intention to treat analysis in randomised trials with missing outcome data. BMJ. 2011;342:d40
17. Wand R, Lagakos SW, Ware JH, Hunter DJ, Drazen JM. Reporting of subgroups analyses in clinical trials. NEJM. 2007;357:2189-94
18. https://www.bapm.org/resources/40-identification-and-management-of-neonatal-hypoglycaemia-in-the-full-term-infant-2017
19. Chatfield A, Caglia JM, Dhillon S, Hirst J, Cheikh Ismail L, Abawi K et al for the International Fetal and Newborn Growth Consortium for the 21st Century (INTERGROWTH-21st). Translating research into practice: the introduction of the INTERGROWTH-21st package of clinical standards, tools and guidelines into policies, programmes and services. BJOG 2013;120 (Suppl.

2): 139–142

Appendix A: Deviations from SAP

This report below follows the statistical analysis plan dated *<insert effective date of latest SAP>* apart from following:

**Section of report not following SAP**	**Reason**
<insert section >	<insert, e.g. exploratory analyses request by TMG>

Appendix B: Trial schema

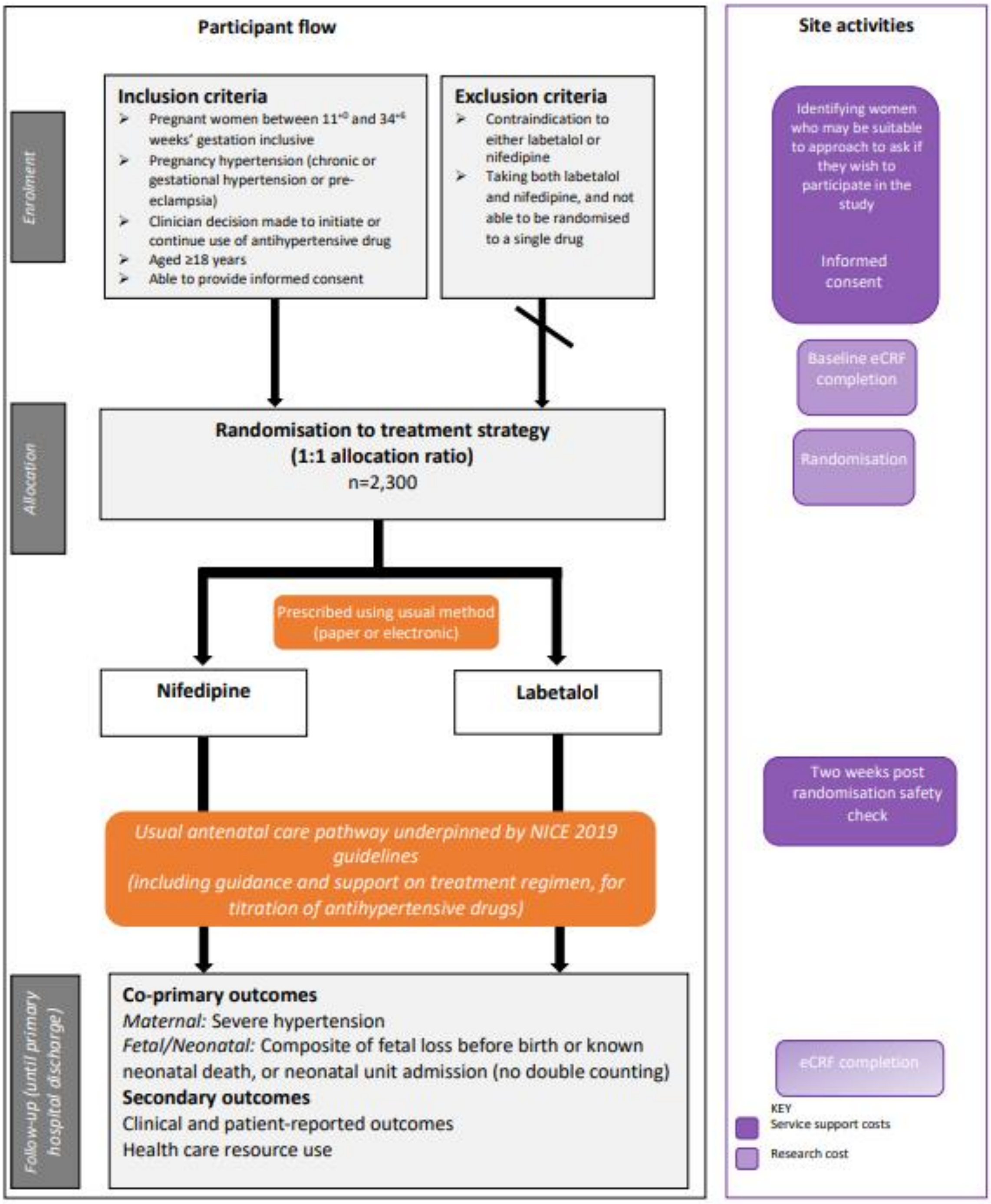

Appendix C: Schedule of assessments

Visit	Screening	Randomisation	Antenatal period	Post delivery
Eligibility check	x			
**Valid informed consent***	x			
**Randomisation and prescription of antihypertensive drug*†**		x		
**Two weeks post enrolment contact***			x	
**Six weeks post enrolment contact (four weekly thereafter)***			x	
Safety reporting (as needed)			x	x
Case note review (safety and other outcomes)				x

***Contact with the participating woman.**
†In randomised cohort only.

Appendix D: Template report

A template report for the final analyses will be provided in a separate document.

Appendix E: Data manipulations

The Trial Statistician will derive all responses from the raw data recorded in the database as follows:

Primary outcomes

Maternal: Severe hypertension (proportion of days with a healthcare professional measured systolic blood pressure reading ≥160mmHg between randomisation and birth)

For any women who have an inpatient stay (including birth) post-randomisation (post-consent for those in the observational cohort) this will be recorded on CRF 8b. Number of days where blood pressure readings were taken during this inpatient stay will be calculated as follows:

If the admission did not include birth (i.e. admissions pre-birth):

(Date of discharge-Date of admission)-(Number of days where no blood pressure recordings taken)

If the admission included birth:

(Date of birth-Date of admission)-(Number of days where no blood pressure recordings taken)

For X inpatient admissions, the total number of days where blood pressure readings were taken will be calculated as:

X_1=
(Number of days where blood pressure readings taken during inpatient admission 1) +
(Number of days where blood pressure readings taken during inpatient admission 2) +
……
+ (Number of days where blood pressure readings taken during inpatient admission X)

For each day during an admission (up to and including the day of birth) if at least one blood pressure reading was taken during that day and the highest systolic blood pressure reading across this day was at or above 160mmHg this will count towards the outcome. If at least one blood pressure reading was taken during that day and the highest systolic blood pressure reading across this day was below 160mmHg this will not count towards the outcome. For X inpatient admissions, the total number of days where the highest systolic blood pressure reading was ≥160mmHg will be calculated as follows:

Y_1=
(Number of days during inpatient admission 1 where highest daily blood pressure was ≥160mmHg) +
(Number of days during inpatient admission 2 where highest daily blood pressure was ≥160mmHg) +
……
+ (Number of days during inpatient admission X where highest daily blood pressure was ≥160mmHg)

For any women who have an outpatient stay post-randomisation (post-consent for those in the observational cohort) this will be recorded on CRF 8a. If a woman had her blood pressure measured ‘in person with a healthcare professional’ at this visit this will count as one day. The total number of outpatient appointments which meet this definition will be regarded as X_2.

For each outpatient stay which meets the criteria above, if the highest recorded systolic blood pressure during the outpatient stay was at or above 160mmHg this will count towards the outcome. If at least one blood pressure reading was taken during the outpatient stay and the highest systolic blood pressure reading across this day was below 160mmHg this will not count towards the outcome. The total number

of outpatient appointments which meet this definition will be regarded as Y_2.

The total number of days where blood pressure readings were taken (B) will be calculated as:

$$B = X_1 + X_2$$

The total number of days where the highest systolic blood pressure reading was ≥160mmHg (A) will be calculated as:

$$A = Y_1 + Y_2$$

Severe maternal hypertension will be defined as:

$$A/B$$

(This value will range between 0 and 1).

Fetal/neonatal: Composite of fetal loss before birth or known neonatal death, or neonatal unit admission involving separation of the baby from the mother between randomisation up to primary hospital discharge or 28 days post-birth, whichever occurs sooner (with no double counting of outcomes).
Neonatal outcomes are collected on CRF 11. A neonate will be regarded as meeting this outcome if they meet any of the three following criteria:

- Fetal loss:
Fetal loss before birth will be defined as either ‘registered as stillbirth’, ‘registered as miscarriage’, ‘registered as termination of pregnancy’ or ‘ectopic’.

- Known neonatal death:
If a baby is ‘registered as a live birth’ and baby dies prior to discharge, time to neonatal death will be calculated as:

$$\text{Time to death}=(\text{Date of death}-\text{Date of delivery})$$

If this is >28 days this will not contribute to the outcome. If this is ≤28 days, this will contribute to the outcome.

- Neonatal admission:
If a baby is ‘registered as a live birth’ and ‘Was baby was admitted to the NNU’=Yes this will contribute to the outcome.

<u>**Secondary outcomes**</u>

Severe maternal hypertension (defined as any episode of severe maternal hypertension (systolic blood pressure ≥160 mmHg between randomisation and birth)) [2]
As per the primary outcome, if A≥1 then a woman will have had at least one episode of severe maternal hypertension (systolic blood pressure≥160mmHg) and will be considered to meet this outcome.

Mean antenatal systolic blood pressure (using highest systolic blood pressure per day as collected for

the primary outcome)
The total number of days where at least one blood pressure reading was taken will be calculated as:

$$B = X_1 + X_2$$

Where X_1 and X_2 are derived as per the maternal co-primary outcome above.

The mean antenatal systolic blood pressure will be calculated as:

$$(Z_1 + Z_2 + Z_3 + \ldots + Z_4)/B$$

Where Z_i is the highest systolic blood pressure reading on the ith day where a blood pressure reading is taken.

Mean antenatal diastolic blood pressure (using highest diastolic blood pressure per day)

The total number of days where at least one blood pressure reading was taken will be calculated as:

$$B = X_1 + X_2$$

Where X_1 and X_2 are derived as per the maternal co-primary outcome above.

The mean antenatal diastolic blood pressure will be calculated as:

$$(V_1 + V_2 + V_3 + \ldots + V_4)/B$$

Where V_i is the highest diastolic blood pressure reading on the ith day where a blood pressure reading is taken.

Proportion of days with an antenatal systolic hypertension blood pressure reading ≥140mmHg
As per derivation of the primary outcome (maternal severe hypertension), with a threshold of ≥140mmHg used as opposed to 160mmHg.

Proportion of days with an antenatal diastolic hypertension blood pressure reading ≥90mmHg
As per derivation of the primary outcome (maternal severe hypertension), where diastolic blood pressure is used (as opposed to systolic blood pressure) with a threshold of ≥90mmHg.

New diagnosis of pre-eclampsia* up to birth
Maternal outcomes are collected on CRF 10. If, 'Did the woman have a diagnosis of pre-eclampsia or superimposed pre-eclampsia'=yes (before birth) this will meet the outcome. The denominator will exclude women who had a diagnosis of pre-eclampsia or superimposed pre-eclampsia at randomisation (or consent of those women in the observational cohort).

Diagnosis of eclampsia up to birth
Maternal outcomes are collected on CRF 10. If 'Did the woman experience any of the following: eclampsia'=yes (before birth) this will meet the outcome.

Diagnosis of Haemolysis, Elevated Liver enzymes, Low Platelets syndrome up to birth
Maternal outcomes are collected on CRF 10. If 'Did the woman have a diagnosis of HELLP/ELLP'=yes

(before birth) this will meet the outcome.

Placental abruption up to birth
Maternal outcomes are collected on CRF 10. If 'Did the woman have a placental abruption'=yes this will meet the outcome.

Severe maternal morbidity (fullPIERS consensus definition [3]) up to birth
Severe maternal morbidity will be defined as a woman experiencing any of the following criteria up to birth: Maternal death, Eclampsia, Glasgow coma score <13, Stroke, Transient ischaemic attack, Cortical blindness or retinal detachment, Posterior reversible encephalopathy, Positive inotropic support, Intramuscular/Infusion of a third parental antihypertensive drug, Myocardial ischemia or infarction, SpO_2 <90%, ≥50% FiO_2 for >1h, Intubation (other than for caesarean section), Pulmonary oedema, Transfusion of any blood product, Platelet count $<50x10^9$ per L, with no transfusion, Hepatic dysfunction, Haematoma or rupture, Acute renal insufficiency, Acute renal failure, Dialysis or Placental abruption. These events are recorded on CRF 10.

Maternal death up to birth
Maternal outcomes are collected on CRF 10. If 'Did the woman die before birth'=yes this will meet the outcome.

Maternal stroke up to birth
Maternal outcomes are collected on CRF 10. If 'Did the woman experience any of the following: stroke=yes (before birth) this will meet the outcome.

Prescription of additional antihypertensive drug(s) up to birth
Prescription data are collected at antenatal inpatient contacts (CRF 8a), antenatal outpatient contacts (CRF8b) and each study contact (two week study contact, six week study contact onwards). At each assessment where prescription data is collected (up to birth), if any prescription includes the woman's allocated antihypertensive drug and an additional antihypertensive drug (which is different to the allocated antihypertensive drug) this will meet the outcome.

Prescription of alternative antihypertensive drug(s) up to birth
Prescription data are collected at antenatal inpatient contacts (CRF 8a), antenatal outpatient contacts (CRF8b) and each study contact (two week study contact, six week study contact onwards). At each assessment where prescription data is collected (up to birth), if any prescription does not include the woman's allocated antihypertensive drug and an alternative antihypertensive drug has been prescribed (which is different to the allocated antihypertensive drug) this will meet the outcome.

Discontinued allocated antihypertensive drug
Prescription data are collected at antenatal inpatient contacts (CRF 8a), antenatal outpatient contacts (CRF8b) and each study contact (two week study contact, six week study contact onwards). At each assessment where prescription data is collected (up to birth), if any prescription does not include the woman's allocated antihypertensive drug this will meet the outcome.

Persistence with allocated antihypertensive (time from randomisation to first discontinuation) up to birth
In those women who discontinue allocated antihypertensive (as defined above). Date of discontinuation will be taken as the date of the first assessment where this event occurred (i.e. date of discharge for an

inpatient stay (unless woman delivered during this visit and then date of birth will be used), date of outpatient appointment, date of two week study contact etc.). Time to discontinuation will be calculated as:

Time to discontinuation (weeks)=[Date of first discontinuation-Date of randomisation]/7

Those who do not discontinue allocated antihypertensive will be censored at birth (if full prescription data known up to birth) or at the last point where prescription data was known.

Undesirable effects of allocated (and other) antihypertensive drug(s) (number of women and number of undesirable effects)
Side effect data are collected at antenatal inpatient contacts (CRF 8a), antenatal outpatient contacts (CRF8b) and each study contact (two week study contact, six week study contact onwards). The following side effects are collected: headache, dizziness, weakness, flushing, heart palpitations, chest pain, lower leg oedema, shortness of breath, rash, itchy skin, jaundice, constipation, diarrhoea, epistaxis, tingling scalp, nasal congestion, tiredness, nausea, epigastric/stomach pain and other.

For each side effect (excluding other), if the side effect is reported at any assessment this will meet the outcome.

Number of incidences of the side effect will be calculated as the number of assessments where the side effect is reported. For women who do not present the side effect at any assessment this will be regarded as 0.

Total number of antenatal hospital inpatient days up to birth
Antenatal inpatient contacts are recorded on CRF 8a. If the admission did not include birth (i.e. admissions pre-birth) then:

$$X_1\text{=(Date of discharge-Date of admission)}$$

If the admission included birth:

$$X_2\text{=(Date of birth-Date of admission)}$$

Total number of antenatal hospital inpatient days is calculated as:

$$X_1 + X_2$$

Treatment satisfaction with allocated antihypertensive drug (version 2.0) [4]

For questions 1-2 and 7-11 code as: Extremely dissatisfied=1, Very dissatisfied=2, Dissatisfied=3, Somewhat Satisfied=4, Satisfied=5, Very satisfied=6 and Extremely satisfied=7.

For questions 4-6 code as: Extremely dissatisfied=1, Very dissatisfied=2, Somewhat dissatisfied=3, Slightly dissatisfied=4, Not at all dissatisfied=5 and Not applicable=5.

Four domains can be calculated as follows:

<u>Global satisfaction score:</u>

If both Q10 and Q11 are completed, then:

Global satisfaction score=([SUM(Q10, Q11)-2]/12)*100)

If either Q10 <u>or</u> Q11 (one missing data item) are missing then:

Global satisfaction score=([Completed item-1]/6)*100)

If both Q10 <u>and</u> Q11 are missing then no score is computed.

<u>Effectiveness score:</u>

If both Q1 and Q2 are completed, then:

Effectiveness score=([SUM(Q1, Q2)-2]/12)*100)

If either Q1 <u>or</u> Q2 (one missing data item) are missing then:

Effectiveness score=([Completed item-1]/6)*100)

If both Q1 <u>and</u> Q2 are missing then no score is computed.

<u>Side effects score:</u>

If Q4, Q5 and Q6 are completed, then:

Side effects score=([SUM(Q4, Q5, Q6)-3]/12)*100)

If either Q4, Q5 <u>or</u> Q6 (one missing data item) are missing then:

Side effects score =([SUM(completed items)-2]/8)*100)

If ≥2 data items are missing then no score is computed.

<u>Convenience score:</u>

If Q7, Q8 and Q9 are completed, then:

Convenience score=([SUM(Q7, Q8, Q9)-3]/18)*100)

If either Q7, Q8 <u>or</u> Q9 (one missing data item) are missing then:

Convenience score =([SUM(completed items)-2]/12)*100)

If ≥2 data items are missing then no score is computed.

For all four domains, scores range from 0-100, where higher scores reflect greater dissatisfaction.

Beliefs about allocated antihypertensive drug [5]
The Beliefs about Medicine Questionnaire (BMQ Specific) is collected at the two week study contact. It is scored as following:

For questions BS1-BS11 code as: Strongly agree=5, Agree=4, Uncertain=3, Disagree=2, Strongly disagree=1

BMQ Specific necessity score=SUM(BS1, BS3, BS4, BS7, BS10)/5

BMQ Specific concerns score=SUM(BS2, BS5, BS6, BS8, BS9, BS11)/6

Scores range from 1-5. Missing data are permitted under the following conditions. For both scores, if <60% of items have been completed (<3 items for the BMQ Specific necessity score, <4 items for the BMQ Specific concerns score) then no score will be computed. If ≥60% of items have been completed the scores will be computed as follows:

SUM(completed items)/(number of completed items)

Adherence to allocated (and other) antihypertensive drug(s) [6]
The Medication Adherence Report Scale (MARS-5) is collected at the two week study contact. It is scored as following:

For questions M1-M5 code as: Always=1, Often=2, Sometimes=3, Rarely=4 Never=5.

MARS-5 score=SUM(M1-M5)/5.

Scores range from 1-5. High scores indicate better adherence with medications. Missing data are permitted under the following conditions. If <60% of items have been completed (<3 MARS-5 items) then no score will be computed. If ≥60% of items have been completed the score will be computed as follows:

SUM(completed items)/(number of completed items)

Mode of onset of birth
Mode of onset of birth is recorded on CRF 10.

Indicated delivery
Mode of onset of birth is recorded on CRF 10. Indicated delivery is defined as either induction of labour, pre-labour rupture of membranes (PROM) with stimulation of labour or pre-labour caesarean section.

New episodes of severe maternal morbidity (fullPIERS consensus definition [3]) post-birth
Severe maternal morbidity will be defined as a woman experiencing any of the following criteria post-birth: Maternal death, Eclampsia, Glasgow coma score <13, Stroke, Transient ischaemic attack, Cortical blindness or retinal detachment, Posterior reversible encephalopathy, Positive inotropic support, Intramuscular/Infusion of a third parental antihypertensive drug, Myocardial ischemia or infarction, SpO_2 <90%, ≥50% FiO_2 for >1h, Intubation (other than for caesarean section), Pulmonary oedema, Transfusion of any blood product, Platelet count $<50x10^9$ per L, with no transfusion, Hepatic dysfunction, Haematoma or rupture, Acute renal insufficiency, Acute renal failure, or Dialysis. These events are

recorded on CRF 10.

Maternal death post-birth
Maternal outcomes are collected on CRF 10. If 'Did the woman die before birth'=yes then this meets the outcome.

Fetal loss prior to 24 weeks' gestation
Neonatal outcomes are collected on CRF 11. If either 'registered as stillbirth', 'registered as miscarriage', 'registered as termination of pregnancy' or 'ectopic' = yes then check if

$$\text{Gestation at delivery} < 24 \text{ weeks}$$

If these criteria are satisfied, this outcome is met.

Fetal loss ≥24+0 weeks' gestation (stillbirth)
Neonatal outcomes are collected on CRF 11. If either 'registered as stillbirth', 'registered as miscarriage', 'registered as termination of pregnancy' or 'ectopic' = yes then check if

$$\text{Gestation at delivery} \geq 24 \text{ weeks}$$

If these criteria are satisfied, this outcome is met.

Known early neonatal death (up to 7 days from birth)
Neonatal outcomes are collected on CRF 11. If baby is 'registered as live birth', check baby's status at discharge. If baby 'died prior to discharge'=yes then calculate time to death as following:

$$\text{Time to death (days)} = \text{Date of death} - \text{Date of delivery}$$

If time to death <7 days this will meet the outcome.
If time to death ≥7 days this will not meet the outcome.

If 'discharged from hospital' or 'transfer of care to another unit'=yes and this occurs prior to 7 days, assume baby is alive up to 7 days and does not meet the outcome.
If 'not discharged-baby still an inpatient'=yes then we will have 28 days follow-up and therefore this does not meet the outcome.

Known late neonatal death (between 7 and up to 28 days from birth)
Neonatal outcomes are collected on CRF 11. If baby is 'registered as live birth', check baby's status at discharge. If baby 'died prior to discharge'=yes then calculate time to death as following:

$$\text{Time to death (days)} = \text{Date of death} - \text{Date of delivery}$$

If time to death <7 days this will not meet the outcome.
If time to death ≥7 days and <28 days this will meet the outcome.
If time to death ≥28 days this will not meet the outcome.

If 'discharged from hospital' or 'transfer of care to another unit'=yes and this occurs prior to 28 days, assume baby is alive up to 28 days and does not meet the outcome.
If 'not discharged-baby still an inpatient'=yes then we will have 28 days follow-up and therefore this

does not meet the outcome.

Neonatal unit admission (separation of baby from mother)
Neonatal outcomes are collected on CRF 11. If 'Was baby admitted to the NNU'=yes this will meet the outcome.

Principal recorded indication for neonatal unit admission
Principle indication for NNU admission is recorded on CRF 11.

Length of stay in neonatal unit (and level of care)
Type of NNU care (NICU, HDU and SCBU) and length of stay in each setting is recorded on CRF 11.

Major congenital abnormality as defined by EUROCAT
Major congenital abnormality is recorded on CRF 11.

Mode of birth (spontaneous vaginal, assisted vaginal, Caesarean section)
Mode of birth is recorded on CRF 11. Caesarean section includes elective pre-labour caesarean section, emergency pre-labour caesarean section and emergency caesarean section in labour.

Indication for mode of birth
Indication for mode of birth is recorded on CRF 11.

Gestational age at birth
Gestational age at delivery will be calculated as follows:

Gestational age (weeks)= 40-(((Expected date of delivery)-(Date of delivery))/7).

Preterm birth (<37 completed weeks' gestation)
Defined as delivery prior to 37 weeks (gestational age at delivery derived as above).

Preterm birth (<32 completed weeks' gestation)
Defined as delivery prior to 32 weeks (gestational age at delivery derived as above).

Birthweight
Exclude any babies delivered ≤20 weeks gestational age. Birth weight is recorded on CRF 11.

Birthweight centile
Exclude any babies delivered ≤20 weeks gestational age. Birth weight will be adjusted for gestational age and sex using the intergrowth standards [19]. Centiles will be produced from this output.

Birthweight small for gestational age (<10th centile for gestational age)
Exclude any babies delivered ≤20 weeks gestational age. Birth weight centiles (from intergrowth standards) will be derived as above. If the birth weight centile is <10 this will meet the outcome definition.

Umbilical arterial pH <7 at birth
Umbilical arterial pH at birth is recorded on CRF 11. Include babies is 'registered as live birth' only. An umbilical arterial pH at birth <7 will meet this outcome.

Apgar score at 5 mins after delivery
Apgar score at 5 mins after delivery is recorded on CRF 11. Include babies is 'registered as live birth' only.

Need for additional resuscitation at birth: intubation in the delivery room, resuscitation drugs or chest compressions
Need for additional resuscitation at birth is recorded on CRF 11. Include babies is 'registered as live birth' only.

Need for respiratory support
Need for respiratory support is recorded on CRF 11. Include babies is 'registered as live birth' only.

Type of respiratory support needed
Type of respiratory support needed is recorded on CRF 11. Include babies is 'registered as live birth' only.

Need for treatment for neonatal hypoglycaemia (in those having blood glucose monitoring)
Include babies where 'Did the baby have their blood glucose monitored on at least one occasion within the first 48 hours after birth'=yes only. If 'Did the baby have treatment for their blood glucose'=yes then this will meet the outcome.

Type of treatment for hypoglycaemia
Type of treatment for hypoglycaemia is recorded on CRF 11.

Lowest blood glucose measurement within the first 48 hours after birth
Lowest blood glucose measurement within the first 48 hours after birth is recorded on CRF 11.

Neonatal seizures
Neonatal seizures is recorded on CRF 11. Include babies is 'registered as live birth' only.

Intracranial haemorrhage
Intracranial haemorrhage is recorded on CRF 11. Include babies is 'registered as live birth' only.

Necrotising enterocolitis
Necrotising enterocolitis is recorded on CRF 11. Include babies is 'registered as live birth' only.

<u>**Process outcomes**</u>

Number of babies in whom blood glucose monitoring was indicated at birth
Indication for blood glucose monitoring is recorded on CRF 11. If any of the following are selected: intrauterine growth restricted (<3rd centile), diabetic mother, mother on beta-blockers e.g. labetalol, preterm (<37 weeks' gestation), clinical indications , under birth weight threshold (for gestation), or other this will meet the outcome.

Those who tick any one of the following: intrauterine growth restricted (<3rd centile), diabetic mother, mother on beta-blockers e.g. labetalol, preterm (<37 weeks' gestation), clinical indications or under birth weight threshold (for gestation) will meet the BAPM criteria [16] for the sensitivity analysis.

Indication for blood glucose monitoring
Indication for blood glucose monitoring is recorded on CRF 11.

Blood glucose test performed
Blood glucose test performed is recorded on CRF 11. If 'Did the baby have their blood glucose monitored on at least one occasion within the first 48 hours after birth'=yes this will meet the outcome.

Adverse events
Recorded on CRF 15.

<u>Exploratory outcomes</u>

Maternal: Severe hypertension (proportion of days with a healthcare professional measured systolic blood pressure reading ≥160mmHg between randomisation and two-week post-randomisation)
As per derivation of the primary outcome (detailed above) but only including blood pressure assessments up to two-weeks post-randomisation.

Severe maternal hypertension (defined as any episode of severe maternal hypertension (systolic blood pressure ≥160 mmHg between randomisation and two-week post-randomisation))
As per derivation of the secondary outcome (detailed above) but only including blood pressure assessments up to two-weeks post-randomisation.

Mean antenatal systolic blood pressure (using highest systolic blood pressure per day as collected for the primary outcome) between randomisation and two-week post-randomisation
As per derivation of the secondary outcome (detailed above) but only including blood pressure assessments up to two-weeks post-randomisation.

Mean antenatal diastolic blood pressure (using highest diastolic blood pressure per day) between randomisation and two-week post-randomisation
As per derivation of the secondary outcome (detailed above) but only including blood pressure assessments up to two-weeks post-randomisation.

<u>Other measures</u>

- **Gestational age at randomisation (weeks)** = 40-(((Expected date of delivery)-(Date of randomisation))/7).

- **Gestational age at consent (weeks) (observational cohort only)** = 40-(((Expected date of delivery)-(Date of consent))/7).

- **Maternal age at randomisation (years)** = (Date of randomisation-date of birth)/365.25.

- **Maternal age at consent (years) (observational cohort only)** = (Date of consent-date of birth)/365.25.

- **BMI**=weight (kg)/height (m)^2

Appendix F: Observational study

For women who are recruited in the observational cohort, analysis methods will broadly follow those outlined for the Giant PANDA trial detailed above with exception to the following:

Treatment groups

Women will be analysed as per what they were prescribed at the time of consent to the observational study (this is recorded on CRF 4 - Maternal Details (pre-Randomisation)). They will be grouped as Nifedipine monotherapy, Labetalol monotherapy or other antihypertensives (which includes any other monotherapy or any combination therapies).

Analysis comparison groups

For outcomes where effect estimates and confidence intervals are required, the comparison groups will be Nifedipine monotherapy vs. Labetalol monotherapy. No formal testing will be performed for the 'other antihypertensive' group.

Outcomes

All outcomes will be measured from the point of consent for women in the observational cohort. All outcomes will follow the data manipulations rules in Appendix E, with the exception of the following outcomes which are redefined as follows:

Prescription of additional antihypertensive drug(s) up to birth

Prescription data are collected at antenatal inpatient contacts (CRF 8a), antenatal outpatient contacts (CRF8b) and each study contact (two week study contact, six week study contact onwards). At each assessment where prescription data is collected (up to birth), if any prescription includes the antihypertensive drug(s) the woman was prescribed at the time of consent and an additional antihypertensive drug (which is different to the antihypertensive drug(s) the woman was prescribed at the time of consent) this will meet the outcome.

Prescription of alternative antihypertensive drug(s) up to birth

Prescription data are collected at antenatal inpatient contacts (CRF 8a), antenatal outpatient contacts (CRF8b) and each study contact (two week study contact, six week study contact onwards). At each assessment where prescription data is collected (up to birth), if any prescription does not include the antihypertensive drug(s) the woman was prescribed at the time of consent and an alternative antihypertensive drug has been prescribed (which is different to the antihypertensive drug(s) the woman was prescribed at the time of consent) this will meet the outcome.

Discontinued antihypertensive drug

Prescription data are collected at antenatal inpatient contacts (CRF 8a), antenatal outpatient contacts (CRF8b) and each study contact (two week study contact, six week study contact onwards). At each assessment where prescription data is collected (up to birth), if any prescription does not include the antihypertensive drug(s) the woman was prescribed at the time of consent this will meet the outcome.

Persistence with antihypertensive (time from consent to first discontinuation) up to birth

In those women who discontinue their antihypertensive drug (as defined above). Date of discontinuation will be taken as the date of the first assessment where this event occurred (i.e. date of discharge for an inpatient stay (unless woman delivered during this visit and then date of birth will be used), date of outpatient appointment, date of two week study contact etc.). Time to discontinuation will be calculated as:

Time to discontinuation (weeks)=[Date of first discontinuation-Date of consent]/7

Those who do not discontinue allocated antihypertensive will be censored at birth (if full prescription data known up to birth) or at the last point where prescription data was known.

Adverse events
No adverse events will be collected for women in the observational study.

Sensitivity analyses
All sensitivity analyses will be conducted with the exception of the per-protocol analyses.

Subgroup analyses
Subgroup analyses will include the same variables considered for the randomised cohort.

A template for reporting data from the observational cohort is given in section 4 of the supplementary template report.
